# Propofol attenuates angiogenesis by activating endoplasmic reticulum stress to suppress TFAP2C-driven VEGFA transcription

**DOI:** 10.1007/s10495-025-02214-w

**Published:** 2026-01-12

**Authors:** Fan Yang, Yi Liu, Hui Li, Xue Shang, Qing Hua, Yun Zhu, Beibei Tao, Zhirong Sun

**Affiliations:** 1 Departmentof Anesthesiology, Shanghai Cancer Center, Fudan University, Shanghai, 200032 China; 2 Department of Oncology, Shanghai Medical College, Fudan University, No. 270 Dong an Road, Shanghai, 200032 China; 3 Shanghai Key Laboratory of Bioactive Small Molecules, Department of Physiology and Pathophysiology, School of Basic Medical Sciences, Fudan University Shanghai Medical College, Shanghai, 200032 China; 4 Department of Anesthesiology, Tongren Hospital, Shanghai Jiao Tong University School of Medicine, Shanghai, China; 5 Department of Anesthesiology, Zhongshan Hospital, Fudan University, Shanghai, 200032 China

**Keywords:** Angiogenesis, Propofol, Transcription factor, VEGFA/VEGFR2, TFAP2C, Endoplasmic reticulum stress

## Abstract

During anesthesia, significant hemodynamic changes often alter the vascular microenvironment and affecting endothelial cell behavior. Propofol, a commonly used intravenous anesthetic, has been widely studied for its role in tumor angiogenesis through tumor cell–derived VEGF–mediated endothelial interactions. However, its direct effects on endothelial cell–mediated angiogenesis in non-malignant diseases such as diabetic retinopathy, diabetic nephropathy, and coronary heart disease remain unclear. To address this gap, we examined the effects of propofol on VEGFA-mediated angiogenesis in vitro and in vivo. Mechanistically, propofol triggers endoplasmic reticulum stress by promoting phosphorylation of PERK and its downstream effector eIF2α, leading to suppressed translation of TFAP2C—a transcription factor critical for endothelial function. Further analysis revealed that TFAP2C directly binds to the VEGFA promoter to activate its transcription, thereby facilitating VEGFA/VEGFR2-dependent angiogenesis. Together, these findings not only broaden the understanding of propofol’s pharmacological profile, but also identify TFAP2C as a novel transcriptional regulator of VEGFA, offering new perspectives for therapeutic targeting of VEGFA-mediated angiogenesis.

**Graphical abstract:**

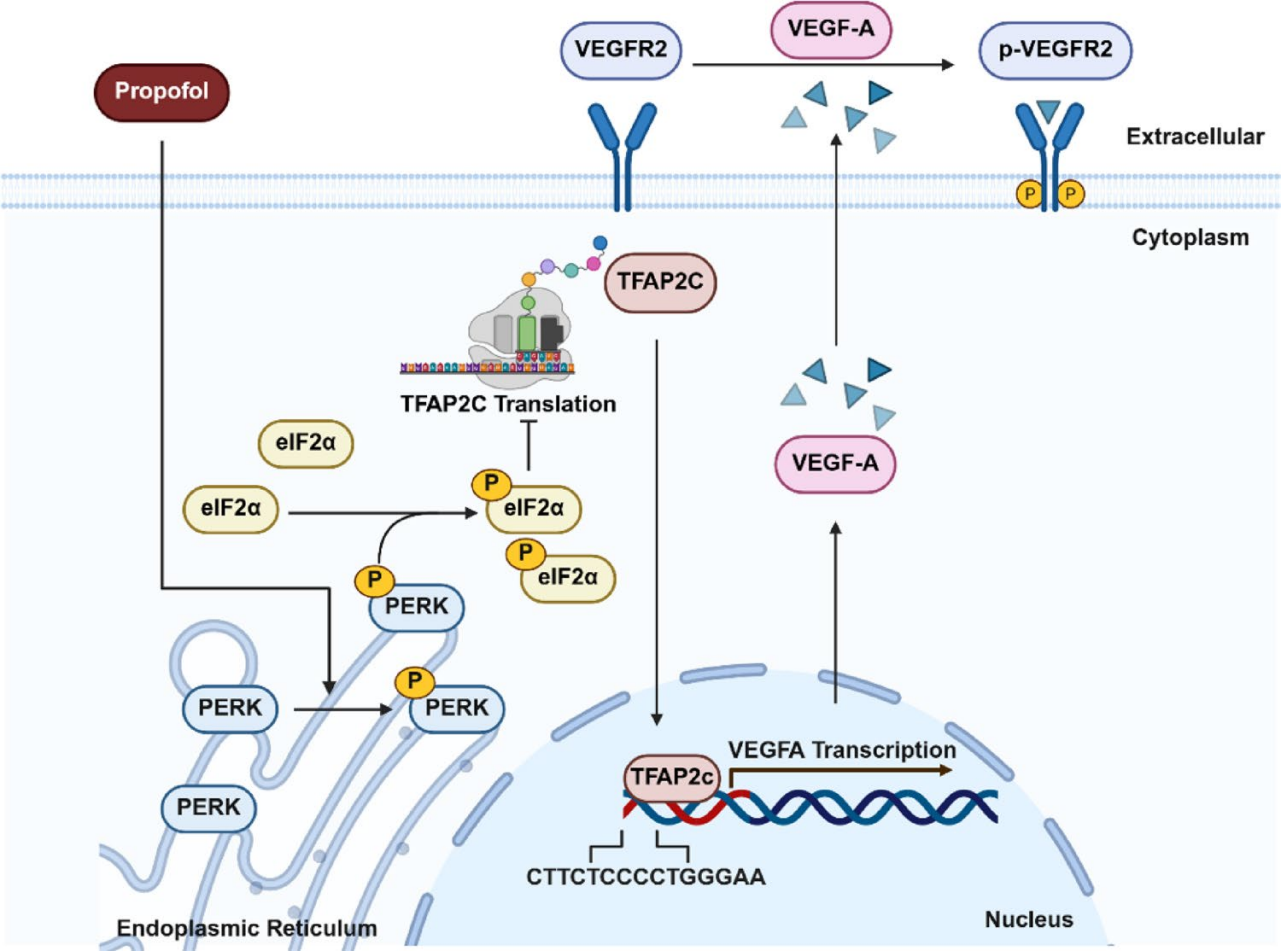

Schematic representation of the mechanism of propofol inhibition of VEGFA/VEGFR2-associated angiogenesis via the PERK/eIF2α/TFAP2C axis.

**Supplementary Information:**

The online version contains supplementary material available at 10.1007/s10495-025-02214-w.

## Introduction

Angiogenesis refers to the formation of new capillaries from pre-existing blood vessels through endothelial cell proliferation, migration, and tube formation [1, 2]. This process is essential for maintaining normal physiological functions and facilitating tissue repair [3]. However, in certain pathological conditions such as malignant tumors [4], diabetic retinopathy [5], rheumatoid arthritis [6], and atherosclerosis [7], angiogenesis can become aberrantly activated, leading to the formation of dysfunctional blood vessels. These structurally abnormal and functionally impaired blood vessels not only fail to provide adequate perfusion but also exacerbate disease progression and pathological damage [8]. Vascular endothelial growth factor (VEGF) plays an important role in endothelial cells (ECs) as an angiogenic factor [9]. VEGFA is the most important angiogenic factor of the VEGF family, and it controls neovascularization through the VEGFR2-mediated signaling pathway [10–15]. Abnormal expression of VEGFA can lead to a variety of diseases, so VEGFA and its signaling pathway have become an important target for anti-angiogenesis therapy [16].

Anaesthesia is often accompanied by significant changes in haemodynamics, such as a decrease in blood pressure and cardiac output, which may affect tissue perfusion and vascular function [17]. The intravenous anaesthetic propofol not only affects the patients’ state of consciousness, but may also indirectly regulate tissue perfusion and microenvironment by altering the haemodynamic state, which in turn may act on ECs [18, 19]. Our previous study found that anaesthetic drugs modulate the inflammatory response, oxidative stress and barrier function of ECs [20–25]. It has also been shown that propofol disrupts the tumor angiogenic microenvironment by inhibiting the secretion of pro-angiogenic factors by tumor cells [26–29]. For example, Wang et al. identified that propofol inhibits the VEGF/VEGFR2 and mTOR/4EBP1 signalling pathway in tumor angiogenesis model in vitro [29].

Previous studies have mainly focused on the role of propofol in modulating VEGF secretion from tumor cells and their interaction with ECs. However, as indispensable participants in angiogenesis, ECs produce endogenous VEGF that plays a crucial role not only in tumors but also in other pathological conditions. Nevertheless, whether propofol directly targets ECs to regulate angiogenesis in broader pathological contexts remains to be fully elucidated, and the underlying mechanisms are not yet clear.

Previous studies have shown that propofol induces activation of endoplasmic reticulum stress (ERS) [30–33]. ERS is the accumulation of misfolded or unfolded proteins in the endoplasmic reticulum when cells are exposed to deleterious stimuli or physiological changes [34]. This process triggers signaling cascades such as activation of protein kinase R (PKR)-like endoplasmic reticulum kinase (PERK) and phosphorylation of eukaryotic initiation factor 2α (eIF2α) downstream of PERK [35–37]. While propofol is known to influence ERS, the role of ERS in angiogenesis and its potential modulation by propofol in ECs remains unexplored.

TFAP2C (also known as AP2γ), a member of the AP2 family of transcription factors, is widely involved in the biological processes of cell proliferation, differentiation and apoptosis [38–41]. Abnormal TFAP2C expression is closely associated with malignant biological behaviors such as proliferation, migration, invasion, and epithelial-mesenchymal transition (EMT) etc., and it has become one of the most important biomarkers for the assessment of tumorigenesis, progression and prognosis [42, 43]. In addition to this, the transcription factor TFAP2C reversed miR − 3656-induced damage to human umbilical vein endothelial cells (HUVECs) by promoting the transcription of Krüppel-like factor 10 (KLF10), implying that TFAP2C may have a potential protective effect on ECs [44]. However, the role of TFAP2C as a transcription factor in angiogenesis remains largely unexplored, and its regulation of VEGFA transcription has not been previously characterized.

In the current study, we investigated the direct effects of propofol on ECs–mediated angiogenesis and further explored its molecular mechanisms in vitro and in vivo. The results indicate that propofol hinders angiogenesis through the inhibition of ECs proliferation, migration, and tube formation. Mechanistically, propofol activates the ER stress–associated PERK/eIF2α pathway, which suppresses TFAP2C translation. Reduced TFAP2C levels in turn decrease its binding to the VEGFA promoter, thereby limiting VEGFA/VEGFR2-dependent angiogenesis. These findings reveal TFAP2C as a novel VEGFA transcription factor and extend the pharmacological profile of propofol beyond anesthesia, offering new avenues to inhibit VEGFA-mediated angiogenesis.

## Materials and methods

### Cell culture

HUVECs were obtained from Zhong Qiao Xin Zhou Biotechnology Co.Ltd.(Shanghai, China), with cells being passaged at a 1:3 ratio. Primary HUVECs were cultured in ECM (ScienCell, USA) supplemented with 10% fetal bovine serum and 1% endothelial cell growth supplement, along with 1% penicillin/streptomycin solution, under 5% CO2 at 37 °C. Passages 3 to 7 of HUVECs were used in all experiments. Human embryonic kidney 293T (HEK 293T) cells were obtained from ATCC (Gibco, USA) and cultured in DMEM medium (Gibco, USA) supplemented with 10% fetal bovine serum at 37 ◦C with 5% CO2. Using dimethyl sulfoxide (DMSO) or PBS as a solvent, propofol (1572503-500 MG, SigmaAldrich, USA), ISRIB (trans- isomer) (HY-12495, MedChemExpress, China), Rapamycin (HY-10219, MedChemExpress, China), MHY1485 (HY-B0795, MedChemExpress, China) and Bevacizumab (HY-P9906, MedChemExpress, China) were prepared as base solution according to the instructions. Propofol was administered to HUVECs at concentrations of 5, 10, 25, and 50 µM. The change of cellular RNA and protein levels were detected after 24 h incubations.

### Lentivirus-mediated gene knockdown

To establish cell lines stably overexpressing TFAP2C, pcDNA3.1-TFAP2C plasmid was designed and synthesized by Guangzhou GeneReal Co. (Guangzhou, China) for TFAP2C gene overexpression, along with a negative control. The pLKO.1-CMV-copGFP-Puro (Genomeditech, Shanghai, China) plasmid was used to generate the TFAP2C shRNA constructs. Lentiviral particles were produced by transfecting the psPAX2 and pMD2.G plasmids into HEK 293T cells using Lipofectamine 2000 (Invitrogen, USA) according to the manufacturer’s protocol. Polybrene (6 µg/mL) was used to enhance lentivirus transfection efficiency, and puromycin (10 µg/mL) was used to select stably expressing cell lines. Transfection efficiency was assessed via Western blot analysis.

### Cell proliferation assay

The viability of cells was detected using Edu Cell Proliferation Kit with Alexa Fluor 555 (Beyotime, C0075). HUVECs were seeded into 24-well plates (3 × 10^5^ cells/well) and treated in accordance with the manufacturer’s instructions. Finally, images were visualized and photographed using Olympus cellSens Entry.

### Wound healing assay

The HUVECs were counted and seeded into a 6-well plate and cultured until 100% confluence and then synchronized with ECM basal medium containing 0.5% fetal bovine serum for 12 h. The monolayer cells were scratched with a plastic pipette; detached cells were washed away 3 times by PBS. Images of the wound were taken at 0 h and 24 h after the scratch and subsequently quantified by ImageJ software.

### Transwell migration assay

The transwell migration assay was performed using 24-well cell culture inserts containing a transparent PET membrane (BD Biosciences, USA). A total of 2 × 105 cells in 200 µL serum-free ECM were added to the upper chamber, and 800 µL ECM supplemented with 10% fetal bovine serum was added to the lower chamber. Following 24 h of incubation, the migrated cells on the bottom of the membrane were fixed with 4% PFA for 20 min and stained with 0.1% crystal violet for further analysis.

### Tube formation assay

Matrigel (Corning, USA) was plated in 96-well plates and incubated at 37 ◦C for 30 min before seeding the HUVECs. The HUVECs were pre-synchronized with ECM basal medium containing 0.5% FBS for 12 h. Then, 1 × 10^4^ cells were counted and seeded into the solidified Matrigel and incubated for 4 h before photographing. Number of branching points and nodes were quantified using the ImageJ software.

### Flow cytometry analysis

Apoptosis was determined using an Annexin V-FITC/PI Apoptosis Detection Kit (Beyotime, Shanghai, China), consistent with previously reported methods. Cells were collected and incubated with 195 µL of binding buffer. Subsequently, 5 µL of Annexin V-FITC and 10 µL of PI were added, and the incubation was carried out for 20 min at room temperature in the absence of light. The analysis of apoptosis was finally conducted using a flow cytometer.

### Caspase-3 activity assay

Caspase-3 activity was measured using a Caspase-3 Activity Assay Kit (Beyotime, Shanghai, China) following the manufacturer’s protocol. Briefly, HUVECs were treated with various concentrations of propofol (0–50 µM) for 24 h and lysed on ice in the supplied lysis buffer for 15 min. After centrifugation at 16,000 × g for 10 min at 4 °C, the supernatants were collected and incubated with the Ac-DEVD-pNA substrate at 37 °C for 1 h. The release of p-nitroaniline (pNA) was measured at 405 nm using a microplate reader. Caspase-3 activity was expressed as the fold change relative to the control group.

### RNA isolation and quantitative real‑time PCR (qRT‒PCR)

RNAs from HUVECs were isolated using TRNzol reagent (Tiangen Biotech, Beijing, China) with reference to the manufacturer’s protocol. The RNAs were reverse transcribed into cDNA by using PrimeScript FAST RT reagent Kit with gDNA Eraser (Takara, Beijing, China), followed by qRT‒PCR. It was conducted as stated in the manual of TB Green^®^ Premix Ex Taq™ II FAST qPCR (Takara, Beijing, China) with an Applied Biosystems 7300 Detection System (Applied Biosystems^®^, CA). The primers used are shown in Table S1.

### Western blot (WB) analysis

Total protein was extracted from treated HUVECs, and protein concentrations were determined using a BCA protein assay kit (Epizyme, Shanghai, China). Equal amounts of protein samples were separated by 10% SDS-PAGE and transferred onto 0.45 μM PVDF membranes (Epizyme, Shanghai, China). Membranes were blocked with 5% non-fat milk for 2 h at room temperature and then incubated with primary antibodies overnight at 4 °C. After incubation with secondary antibodies, chemiluminescent signals were detected using a chemiluminescence imaging system and quantified with ImageJ software. The antibodies used are shown in Table S2.

### Immunofluorescence (IF) staining

HUVECs were seeded into 35 mm glass-bottom culture dishes and fixed with 4% paraformaldehyde. After washing three times with PBS, the cells were blocked with 10% BSA solution at room temperature for 2 h. Primary antibodies were then added simultaneously and incubated for 12 h at 4 °C. After incubation with fluorescent secondary antibodie for 2 h, the cells were labeled with 4’,6-diamidino-2-phenylindole (DAPI). Confocal laser scanning microscopy was performed using a confocal microscope. The antibodies used are shown in Table S2.

### Prediction of VEGFA-related transcription factors

To identify potential transcription factors regulating VEGFA transcription, we analyzed the VEGFA promoter region using four public databases: ENCODE, CHEA, GTRD, and JASPAR. Each database was queried independently for transcription factors predicted or experimentally validated to bind to this region. Candidate transcription factors were defined as those present in the intersection of all four databases, ensuring robust prediction.

### Luciferase reporter assay

To examine the influence of TFAP2C on the activation of the VEGFA promoter, the VEGFA promoter or its truncated variants were inserted upstream of the luciferase reporter gene in the pGL3.0 Basic vector (GenePharma, Shanghai, China), as previously described in studies examining VEGF promoter activity. Stable overexpression or knockdown of HEK 293T cells and their control cells (1 × 10^5^) were seeded in a 24-well plate and cultured for 24 h. Then, cells were co-transfected with the wide-type or mutant luciferase plasmids, pRL-TK plasmid using Lipo3000TM transfection reagent. After transfection for 48 h, the luciferase activity was quantified using the Dual Luciferase Reporter Gene Assay Kit (Promega, USA). The measured Firefly luciferase activity was normalized against the Renilla luciferase activity as an internal control.

### Chromatin Immunoprecipitation (ChIP)—qPCR

ChIP was performed using the ChIP assay kit (GeneCreate Biotechnology, Wuhan, China) according to the manufacturer’s instruction. Briefly, HUVECs (1 × 10^6^ cells) were cross-linked in 1% formaldehyde for 10 min at 37 °C, and then glycine solution was added to stop the reaction. After washing with pre-cold PBS buffer (supplemented with 1 mM PMSF), cells were centrifuged and lysed with SDS lysis buffer containing 1 mM PMSF. The chromatin was ultra-sonicated to fragments (200–500 bp) 10 times with 10 s ultra-sonication at 10 s intervals. The lysates were subsequently incubated with IgG or ChIP-grade antibody against TFAP2C at 4 °C overnight and then incubated with Protein A + G Agarose/Salmon Sperm DNA at 4 °C for 3 h. After washing with low salt and high salt buffer, elution, and reverse cross-linking, the DNA was added with EDTA, Tris pH 6.5, and proteinase K at 45 °C for 1 h and then was purified for qPCR analysis. The primer sequences used for ChIP-qPCR assay in the VEGFA promotor region were provided in Table S3, while the antibodies were listed in Table S2.

### Protein half-life assays

The protein synthesis inhibitor cycloheximide (CHX, 100 µg/ml, SigmaAldrich) was used to treat HUVECs and evaluate protein stability. Total proteins were extracted from cells at 0-, 20-, 40-, and 60-min post CHX administration. Western blot was carried out to evaluate TFAP2C protein levels.

### Puromycin intake assay

HUVECs were exposed to 50 μM propofol for a duration of 24 h. After 24 h, cells were incubated by using puromycin with final concentration of 10 µM for 30 min at 37 °C. After incubation, the levels of puromycin were detected by Western blot and immunofluorescence staining with an anti-puromycin antibody. The antibodies used in this study are listed in Table S2.

### Matrigel plugs assay in vivo

All in vivo experiments were performed in 6-week-old C57BL/B6 female mice. The mice were housed in a room with controlled temperature and a 12-hour light/dark cycle, with ad libitum access to food and water. The animal protocols were approved by the IACUC of Shanghai Model Organisms Center, Inc (approval number: 2024-0072), ensuring compliance with the Guide for the Care and Use of Laboratory Animals (8th edition, National Academies Press), as stipulated in our contractual agreements.

For in vivo evaluation of angiogenesis, Matrigel plugs assay was performed in 6-week-old C57BL/B6 female mice. Mice were anesthetized and injected subcutaneously in both flanks with 0.5 mL ice-cold Matrigel (Corning, USA) mixed with 60U/ml heparin. The mice were randomly divided into control group, propofol group, propofol + ISRIB group, propofol + ISRIB + Bevacizumab group, propofol + oeTFAP2C group, propofol + oeTFAP2C + Bevacizumab group and propofol + rmVEGFA group. Mice were anesthetized and injected with 0.5 mL of ice-cold Matrigel (Corning Incorporated, USA) mixed with 60U/ml heparin subcutaneously in both abdomens. For the propofol group, 30 mg/kg of propofol was injected daily through the tail vein, and the control group was injected with an equivalent dose of 0.1% DMSO solution. Additionally, the propofol + ISRIB group was administered an additional 0.25 mg/kg of ISRIB daily via intraperitoneal injection. The propofol + oeTFAP2C group received a daily tail vein injection of lentiviral overexpression of TFAP2C concentrated 2 × 107 PFU / 200 µL. Additionally, the propofol + ISRIB + bevacizumab group and the propofol + oeTFAP2C + bevacizumab group received bevacizumab at 5 mg/kg daily via intraperitoneal injection. Recombinant mouse VEGFA (rmVEGFA) was prepared in PBS containing 0.1% bovine serum albumin (BSA) to prevent protein adsorption. propofol + rmVEGFA group mice received daily tail vein injections of VEGFA at a dose of 100 µg/kg for a total of 1 injection per day. Control mice received an equal volume of PBS containing 0.1% BSA. All injections were performed under sterile conditions, and mice were monitored daily for health status and injection site reactions.

After 1 week, the Matrigel plugs were collected and measured for hemoglobin (Hb) content (Beyotime, Shanghai, China) or made paraffin-embedded.

### Immunohistochemical (IHC) staining

For Matrigel plugs collected from the C57BL/B6 mice, Matrigel plugs were embedded in paraffin and cut into 4 μM sections. After the sections were dewaxed and hydrated, the tissues were treated with 3% H_2_O_2_ to block the presence of endogenous peroxidase. Antigen repair was performed using citrate buffer (pH 6.0) or TE buffer (pH 9.0). To prevent nonspecific binding, sections were blocked with normal goat serum. Primary antibodies were then applied to the tissues and incubated at 4 °C overnight. Diaminobenzidine was used for colour development and hematoxylin was used as a counterstain. Images were acquired under a light microscope and positively stained cells were analysed. The antibodies used are shown in Table S2.

### Hematoxylin & Eosin (H&E) staining

The Matrigel plugs embedded in paraffin were deparaffinized and stained with haematoxylin for 10 min. After treatment with hydrochloric acid alcohol solution and ammonium hydroxide for 30 s, the samples were stained with eosin for 3 min. Increased concentrations of alcohol were used to dehydrate the sections. Next, the sections were treated with xylene three times for 3 min each. Finally, neutral balsam was used for section mounting.

### Statistical analyses

Data were reported as mean ± standard deviation (SD) from at least three independent experiments unless otherwise specified. Data were analyzed by two-tailed unpaired Student t test between two groups and by oneway ANOVA followed by Bonferroni test for multiple comparisons. Statistical analysis was carried out using SPSS 16.0 for Windows. All statistical tests were two sided. significance was defined as follows: * *P* < 0.05; ** *P* < 0.01; ns, not significant.

## Results

### Propofol suppresses proliferation, migration and tube formation of HUVECs

**Fig. 1:**
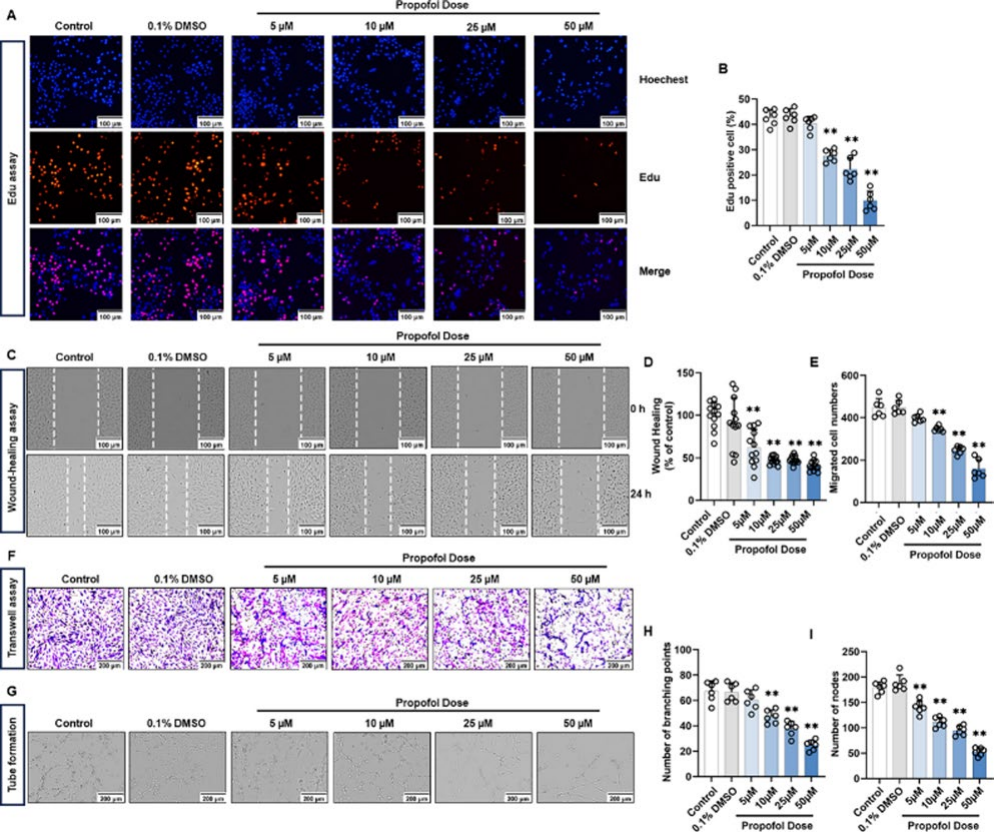
Propofol inhibits the proliferation, migration and tube-formation of HUVECs in a dose dependent manner in vitro. HUVECs cell lines were treated with different concentrations of propofol (5–50 µM), while a blank control and solvent control were set up. **A**,** B** Representative Edu proliferation assay images of HUVECs (**A**) and quantification (**B**). **C**,** D** Representative wound healing migration images of HUVECs (**C**) and quantification (**D**). **E**,** F** Representative transwell migration images of HUVECs (**E**) and quantification (**F**). **G-I** Representative tube-formation images of in vitro angiogenesis (**G**) and quantification of the branches points (**H**) and nodes (**I**). All data are presented as the mean ± SD from 6–12 independent experiments. *P < 0.05, **P < 0.01

To evaluate the effect of propofol on HUVECs, we incubated the cells with medium alone (control), 0.1% DMSO (solvent), and different concentrations of propofol (5–50 µM) for 24 h, and subsequently assessed cell proliferation, migration, and tube formation. The results of Edu assay showed that propofol treatment ranging from 10 to 50 µM inhibited the proliferation of HUVECs, while 5 µM propofol did not produce any significant effect on cell proliferation (Fig. 1A, B). To determine whether the reduced EdU incorporation was due to cytotoxicity, we evaluated apoptosis in HUVECs exposed to propofol. Flow cytometry analysis with Annexin V–FITC/PI staining showed no significant differences in apoptosis among the control, solvent, and 5–50 µM propofol-treated groups (Fig. S1A). Quantitative analysis demonstrated that the proportions of early apoptotic, late apoptotic, and total apoptotic cells remained comparable across all groups (Fig. S1B–D). Consistently, Caspase-3 activity assays revealed no significant change after 24 h of treatment with propofol (Fig. S1E). These results indicate that propofol does not induce apoptosis or cytotoxicity in HUVECs, suggesting that its inhibitory effect on proliferation reflects cytostatic rather than cytotoxic mechanisms.

The wound healing assay showed that 5–50 µM propofol inhibited the migration of HUVECs (Fig. 1C, D). As for the transwell assay, 10–50 µM propofol inhibited the migration of HUVECs per field of view compared with the control (Fig. 1E, F). These data demonstrate that propofol inhibits HUVECs migration in a concentration-dependent manner. In Fig. 1G, HUVECs from control and solvent groups showed robust and well-developed tube structures, but the addition of propofol inhibited tube formation in a concentration-dependent manner. Inhibition of tube formation by propofol is manifested by a reduction in the number of branching points (Fig. 1H) and the number of nodes (Fig. 1I). In conclusion, these data demonstrate that propofol inhibits the proliferation, migration, and tube formation of HUVECs in vitro.

### Exogenous VEGFA reverses the anti-angiogenic effect of Propofol

**Fig. 2:**
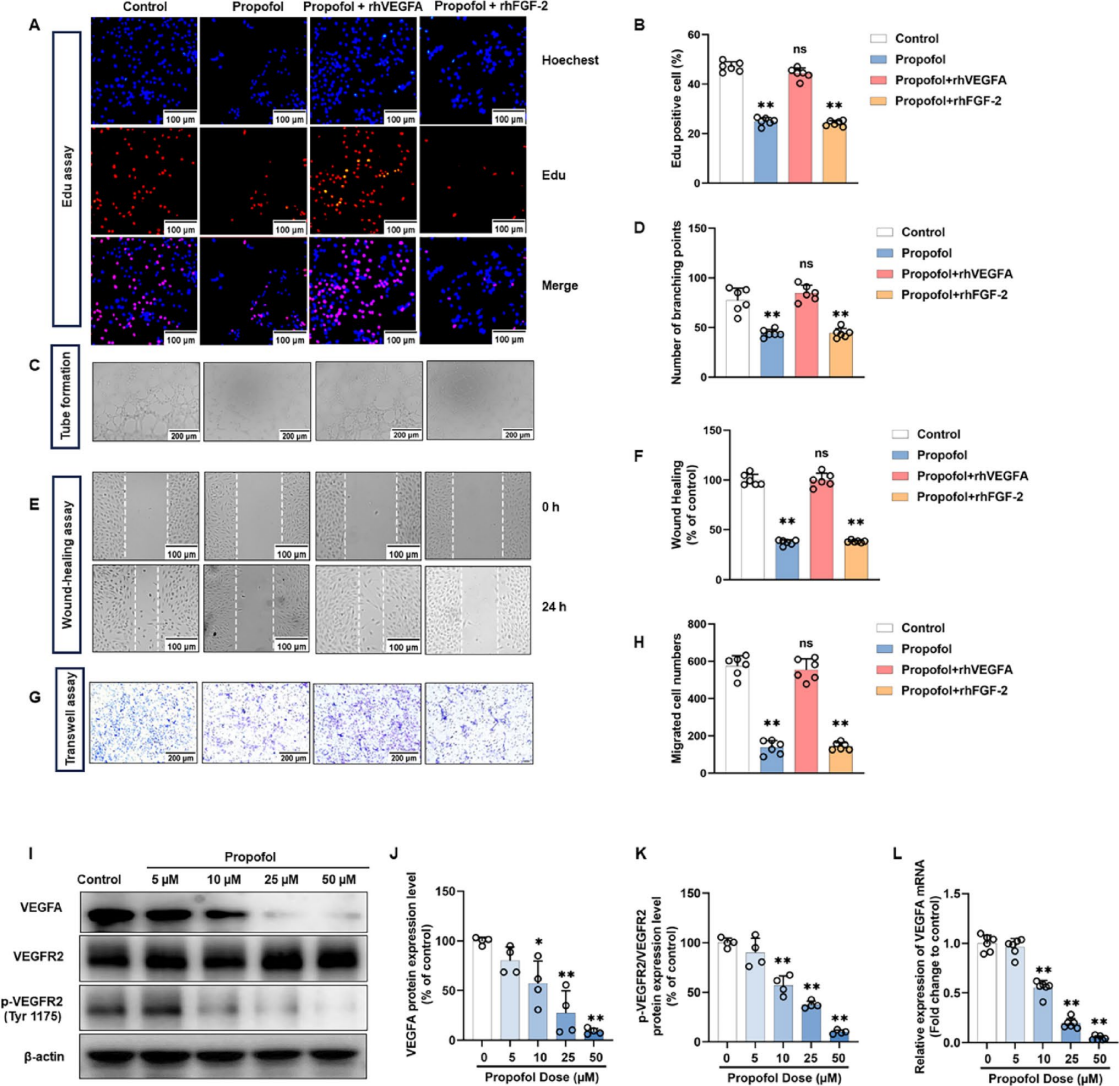
Propofol inhibits angiogenesis through the VEGFA/VEGFR2 signaling pathway in vitro. HUVECs cell lines were treated with 0–50 µM propofol or 10 ng/mL rhVEGFA or rhFGF-2 for 24 h and a solvent control was set up. **A**,** B** Representative Edu proliferation assay images of HUVECs (**A**) and quantification (**B**). **C**,** D** Representative tube-formation images of in vitro angiogenesis (**C**) and quantification of the branches points (**D**). **E**,** F** Representative wound healing migration images of HUVECs (**E**) and quantification (**F**). **G-H** Representative transwell migration images of HUVECs (**G**) and quantification (**H**). **I** Western blot was performed to detect protein expression after treating HUVECs with different concentrations of propofol (0–50 µM) for 24 h. **J**,** K** Densitometric ratios for protein expression in (**I**) were quantified. **L** qPCR was performed to detect VEGFA mRNA expression after treating HUVECs with different concentrations of propofol (0–50 µM) for 24 h. All data are presented as the mean ± SD from 4–6 independent experiments. *P < 0.05, **P < 0.01

VEGFA and FGF2 are the two most potent angiogenic factors [45]. To investigate whether the inhibitory effect of propofol on angiogenesis is mediated through the VEGFA or FGF2, we performed in vitro rescue experiments using 10 ng/mL recombinant human VEGFA (rhVEGFA) or 10 ng/mL recombinant human FGF2 (rhFGF2). The EdU assay showed that treatment with 50 µM propofol for 24 h significantly inhibited the proliferation of HUVECs. Supplementation with rhVEGFA markedly restored the proliferative capacity, whereas rhFGF2 failed to exert any rescuing effect (Fig. 2A, B). In the tube formation assay, control HUVECs formed extensive and well-organized tubular networks. This ability was markedly suppressed by 50 µM propofol, but rhVEGFA supplementation partially reversed the inhibition, as evidenced by increased numbers of branching points. In contrast, rhFGF2 did not restore tube formation (Fig. 2C, D). Similarly, wound healing and transwell assays revealed that 50 µM propofol markedly suppressed HUVECs migration, and this inhibitory effect was partially rescued by rhVEGFA but not by rhFGF2 (Fig. 2E, H).

Collectively, these data demonstrate that exogenous rhVEGFA, but not rhFGF2, can counteract the inhibitory effects of propofol, indicating that propofol suppresses angiogenesis predominantly by targeting the VEGFA rather than FGF2.

To confirm the pro-angiogenic effect of VEGFA, we treated HUVECs with 10 ng/mL rhVEGFA in vitro. The EdU assay showed that rhVEGFA significantly promoted HUVECs proliferation compared with untreated controls (Fig. S2A, B). In the tube formation assay, rhVEGFA-treated HUVECs formed more extensive and well-organized tubular networks, as indicated by an increased number of branching points relative to controls (Fig. S2C, D). Consistently, wound healing and transwell assays demonstrated that rhVEGFA markedly enhanced HUVEC motility (Fig. S2E, H). These results confirm VEGFA as a positive control for endothelial proliferation, migration, and tube formation.

### Propofol downregulates the VEGFA/VEGFR2 signaling cascade

To determine the molecular mechanism by which propofol controls angiogenesis, we examined changes in the levels of the VEGFA/VEGFR2 pathway in propofol-treated HUVECs. The results indicated that treatment with 10–50 µM propofol for 24 h significantly suppressed both the protein and mRNA expression levels of VEGFA, compared to the control group (Fig. 2I, L). While 5 μM propofol did not significantly inhibit the expression level of VEGFA (Fig. 2I, L). Meanwhile, the level of VEGFR2 phosphorylation (Tyr1175) downstream of VEGFA was also inhibited after propofol treatment (Fig. 2I, L). Therefore, the inhibition of angiogenesis by propofol may mainly involve the inhibition of the VEGFA/VEGFR2 signaling pathway, thus inhibiting the proliferation, migration and tube formation of HUVECs.

### TFAP2C acts as a positive transcriptional regulator of VEGFA

Considering that both mRNA and protein levels of VEGFA were altered following propofol treatment, we hypothesized that propofol could impact the transcriptional levels of VEGFA. Initially, we considered several well-known transcription factors reported to regulate VEGFA expression, including HIF-1α [46–49], SP-1 [50–52], AP-1 [53–55] and NF-κB [56–58]. However, propofol treatment did not significantly alter the protein levels of these classical regulators (Fig. S3A, D), suggesting that the observed changes in VEGFA expression were unlikely mediated through these pathways. This prompted us to explore other potential transcription factors that might be responsible for propofol-induced VEGFA regulation.

**Fig. 3:**
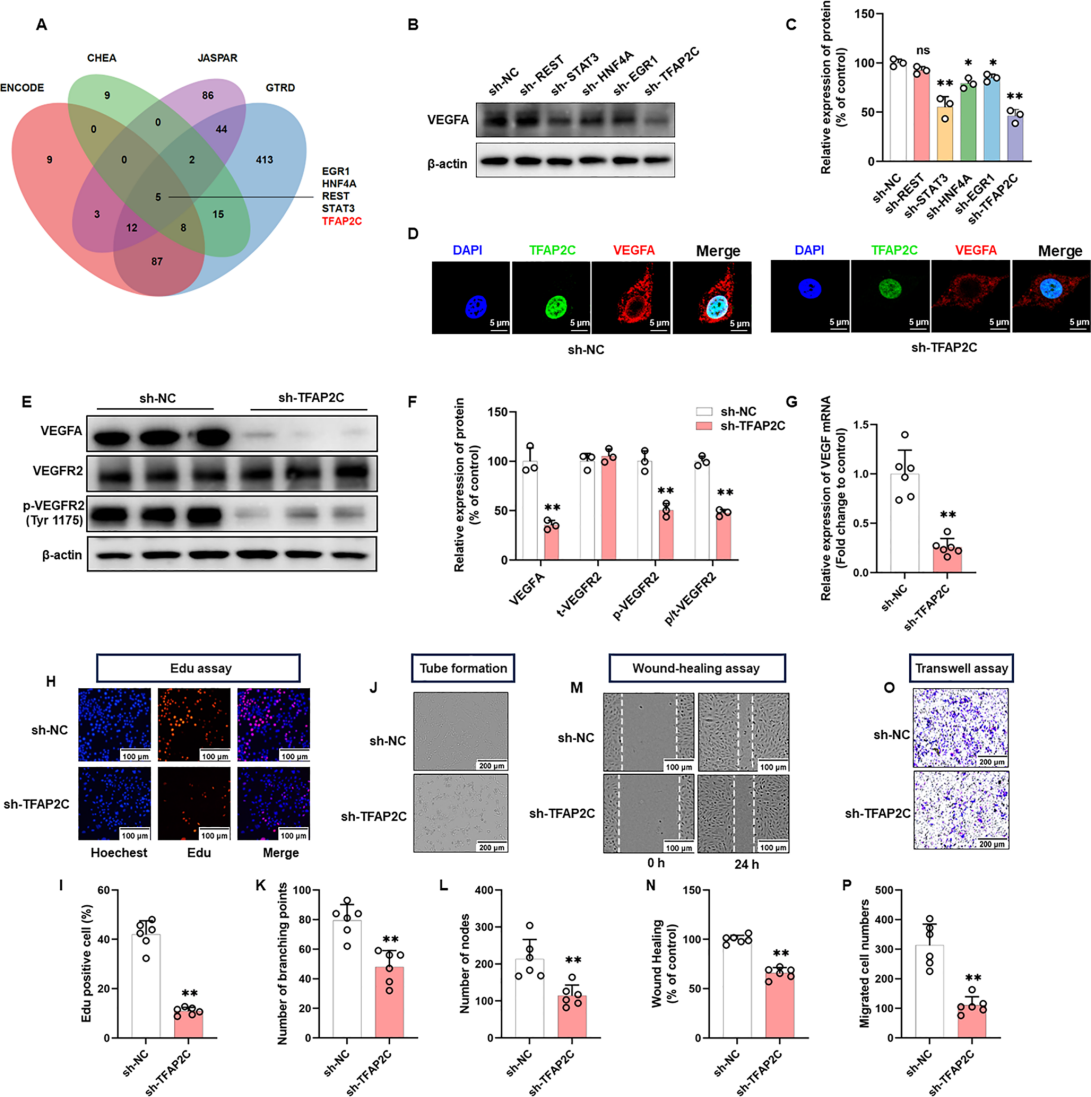
Candidate transcription factors TFAP2C regulates the VEGFA/VEGFR2 signalling pathway and angiogenesis in vitro. **A** Multi-database joint analysis predicts five candidate transcription factors of VEGFA. **B** Western blot analysis shows that VEGFA protein expression levels were observed knocked down for five candidate transcription factors. **C** Quantification of the protein expression of VEGFA represented in (**B**). **D** Representative photomicrographs showing the expression and distribution of TFAP2C and VEGFA in HUVECs with or without TFAP2C knockdown. **E** Western blot analysis showed that decreased levels of VEGFA protein expression and reduced levels of VEGFR2 phosphorylation were observed in TFAP2C knockdown HUVECs cells. **F** Quantification of the protein expression of VEGFA, total-VEGFR2 and p-VEGFR2 (Tyr1175) represented in (**E**). **G** Quantification of the mRNA expression of VEGFA after TFAP2C knockdown. **H**,** I** Representative Edu proliferation assay images of HUVECs (**H**) and quantification (**I**). **J-L** Representative tube-formation images of in vitro angiogenesis (**J**) and quantification of the branches points (**K**) and nodes (**L**). **M**,** N** Representative wound healing migration images of HUVECs (**M**) and quantification (**N**). **O**,** P** Representative transwell migration images of HUVECs (**O**) and quantification (**P**). All data are presented as the mean ± SD from 3–6 independent experiments. *P < 0.05, **P < 0.01

Consequently, we searched the ENCODE, CHEA, GTRD, and JASPAR databases for transcription factors that might activate VEGFA transcription. This led to the identification of five candidate: EGR1, HNF4A, REST, STAT3 and TFAP2C (Fig. 3A). We knocked down each of these five genes and observed VEGFA protein expression. Our results found that except for REST, all candidate proteins were reduced to different degrees (Fig. 3B, C). Among them, STAT3 and TFAP2C showed the most obvious degree of reduction. Given that the transcriptional regulation of VEGFA by STAT3 has been reported [56], TFAP2C was chosen as the next step of the study.

To evaluate the knockdown and overexpression efficiency of TFAP2C in HUVECs, western blot analysis was performed. Among the three designed shRNAs targeting TFAP2C, shTFAP2C-2 exhibited the most pronounced and stable silencing effect, and was therefore selected for subsequent experiments (Fig. S3E). In contrast, TFAP2C protein levels were markedly increased in cells transfected with oe-TFAP2C compared with control and oe-NC groups (Fig. S3F). These findings confirmed the successful establishment of TFAP2C knockdown and overexpression models in HUVECs.

Immunofluorescence revealed that TFAP2C was mainly localized in the nucleus, whereas VEGFA was mainly localized in the cytoplasm, and knockdown of TFAP2C significantly reduced the expression level of VEGFA (Fig. 3D). Western blot (Fig. 3E, F) and qRT – PCR (Fig. 3G) results showed that knockdown of TFAP2C significantly reduced the activation of VEGFA/VEGFR2 signaling pathway. These results indicate that TFAP2C positively regulates VEGFA expression in HUVECs, suggesting a potential role of TFAP2C in modulating angiogenic activity.

### TFAP2C knockdown inhibits angiogenesis in vitro

Ren et al. reported that deletion of the transcription factor TFAP2C was associated with ECs dysfunction in hypertension [44]. Therefore, we generated a TFAP2C knockdown HUVECs to validate the role of TFAP2C in angiogenesis. We assayed cell proliferative activity with the Edu assay, cell migration with the wound healing and transwell assay, and cell lumen forming ability with the tube formation assay. The results of Edu assay showed that cell proliferative activity was significantly decreased in HUVECs with knockdown of TFAP2C (Fig. 3H, I). TFAP2C knockdown inhibited HUVECs tube formation, which was mainly manifested as reduction in the number of branching points and the number of nodes (Fig. 3J, L). Wound healing assay showed that scratch healing was significantly slowed down in TFAP2C low-expressing cells compared with control cells (Fig. 3M, N). In addition, transwell assay showed that TFAP2C knockdown significantly inhibited the migration ability of HUVECs (Fig. 3O, P).

These results indicate that TFAP2C, a candidate gene involved in the regulation of VEGFA transcription, not only positively modulates the VEGFA/VEGFE2 signaling pathway but also enhances proliferation, migration, and angiogenesis in HUVECs in vitro.

### TFAP2C binds to the VEGFA promoter sequence

**Fig. 4:**
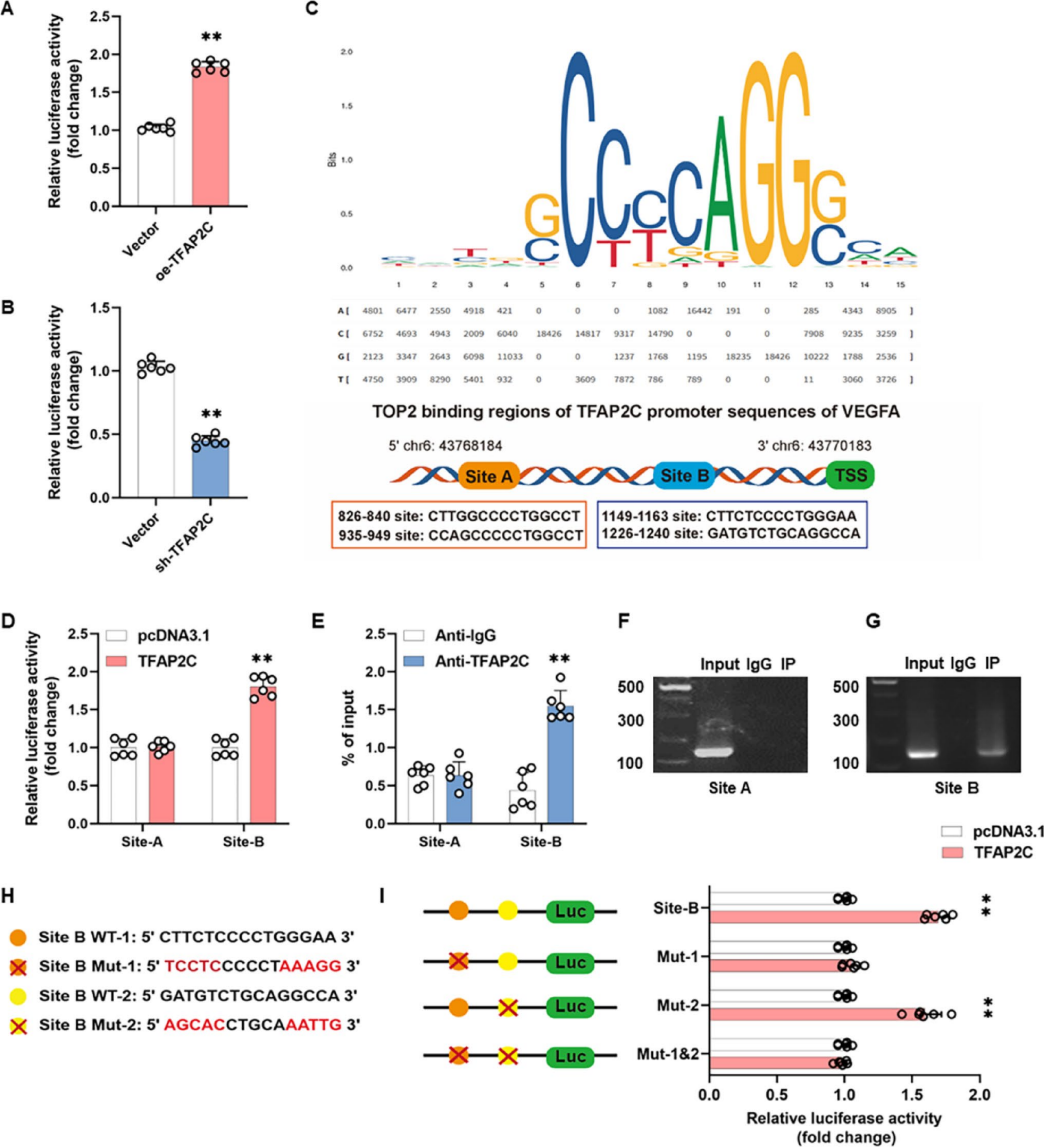
TFAP2C binds to VEGFA promoter sequences. **A**,** B** Luciferase reporter gene studies of the TFAP2C and VEGFA promoters in HEK 293T cells. Cells were cotransfected with VEGFA promoter sequence plasmid (2000 bp) and TFAP2C overexpression (**A**) or knockdown (**B**) plasmid plasmid. **C** JASPAR database predicts putative TFAP2C binding sites identified in the promoter region of VEGFA gene. **D** Luciferase reporter gene studies of TFAP2C and VEGFA promoter in HEK 293T cells. Cells were co-transfected with a plasmid with a truncated VEGFA promoter sequence (Site A and Site B) and a TFAP2C overexpression plasmid. **E** RT-qPCR of the ChIP products confirmed the binding capacity of TFAP2C to the VEGFA promoter. **F-G** Binding of ChIP to PCR provides evidence that TFAP2C binds to Site A in the promoter region of the VEGFA gene. **H** Schematic of Site-B mutation design. **I** Dual-luciferase reporter assays showing that the site mutation abrogated capacity of TFAP2C to act on the VEGFA promoter region. The red “X” within the binding regions indicates altered TFAP2C binding sequences. All data are presented as the mean ± SD from 6 independent experiments. *P < 0.05, **P < 0.01

Based on the above research findings, TFAP2C emerged as a potential transcription factor regulating VEGFA and was selected for further study because of its important role in angiogenesis. To construct a reporter gene plasmid, the 2000 bp sequence upstream of the VEGFA transcription start site (TSS) (Table S3) was amplified and inserted upstream of the firefly luciferase reporter gene. To reduce the influence of intrinsic variability factors on experimental accuracy, the renilla luciferase gene was constructed as a control plasmid. The TFAP2C overexpression or knockdown plasmid was co-transfected with the reporter gene plasmid and control plasmid to detect the expression of luciferase. Dual-luciferase reporter gene assay showed that overexpression of TFAP2C increased luciferase activity (Fig. 4A), whereas knockdown of TFAP2C decreased luciferase activity (Fig. 4B). JASPAR database was used to predict potential TFAP2C-binding sites in the VEGFA promoter. Starting at 2000 bp before the VEGFA TSS, four predicted TFAP2C-binding sites (826–840 bp, 935–949 bp, 1149–1163 bp, 1226–1240 bp) were present in this genomic segment (Fig. 4C). We grouped the four sequences with similar sites and designed them as Site-A (826–949 bp) and Site-B (1149–1240 bp), as shown in Fig. 4C. The dual-luciferase reporter gene assay confirmed that TFAP2C is recruited to the Site-B (1149–1240 bp) of the VEGFA promoter region, rather than the Site-A (826–949 bp, Fig. 4D). ChIP-qPCR experiments similarly showed that TFAP2C binds to the Site-B site in the chromatin region of the VEGFA promoter, but not to the predicted Site-A site (Fig. 4E–G). Given that there are two potential binding sites (1149–1163 bp, 1226–1240 bp) in the Site-B site, we designed point mutations at two potential sites (Mut-1 and Mut-2), as well as simultaneous mutations at two sites, Mut-1 & Mut-2 (Fig. 4H). The luciferase reporter gene plasmids, which contain wild-type (Site-B), mutant (Mut-1 or Mut-2), and co-mutant (Mut-1 and Mut-2) variants, were cloned into the pcDNA3.1 vector. The results of the dual luciferase reporter gene assay showed that mutations in the Mut-1 and Mut-1 & Mut-2 sequences reversed the elevated luciferase activity caused by overexpression of TFAP2C, whereas the luciferase activity of the Mut-2 sequence was unaffected (Fig. 4I). It is suggested that the 1149–1163 bp site of the VEGFA promoter is a binding region for TFAP2C binding.

### Propofol inhibits translation of TFAP2C mRNA into protein

**Fig. 5:**
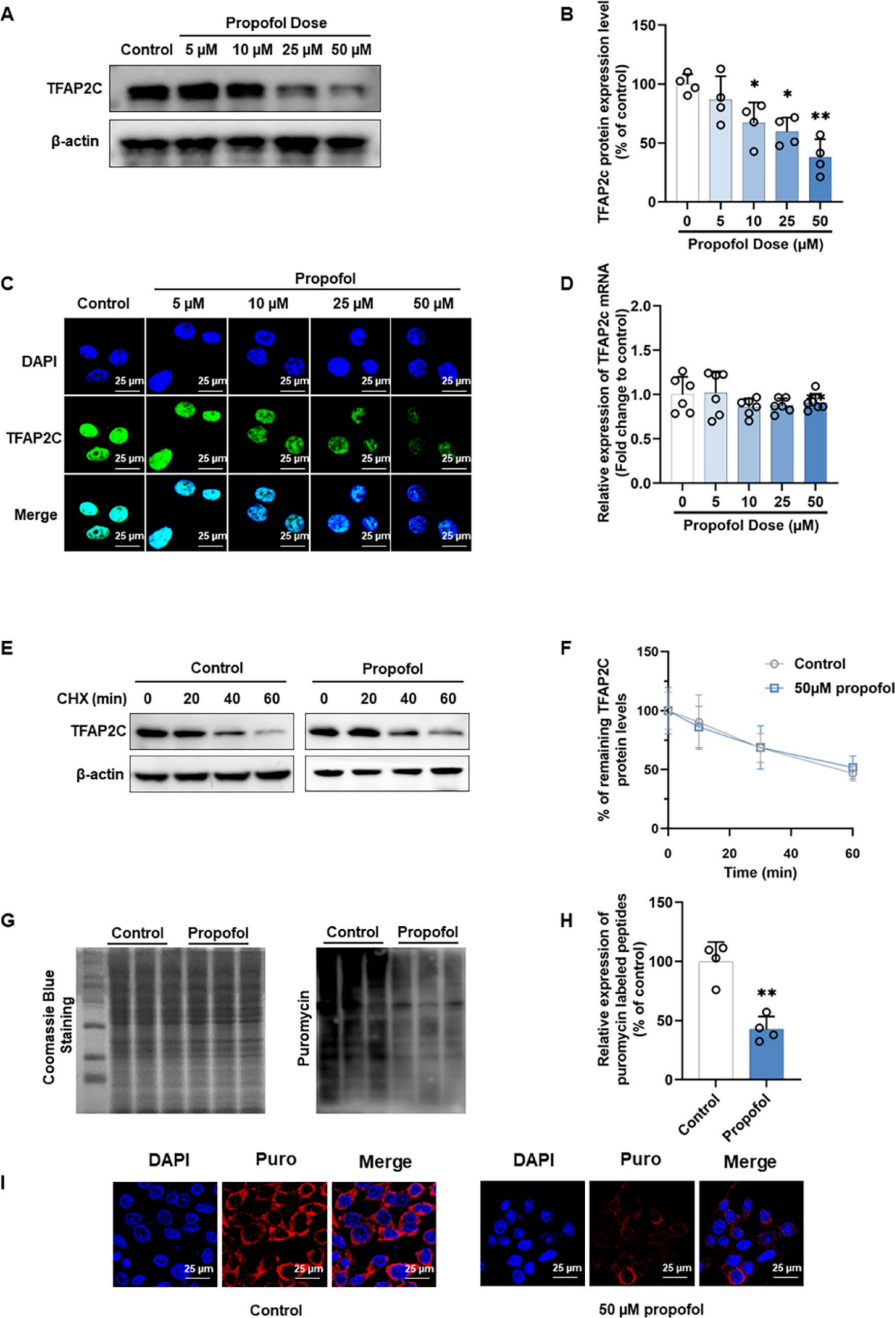
Propofol reduces TFAP2C protein expression from the translational level. **A** Western blot was performed to detect TFAP2C protein expression after treating HUVECs with different concentrations of propofol (0–50 µM) for 24 h. **B** Densitometric ratios for TFAP2C expression in (**A**) were quantified. **C** Representative photomicrographs showing the expression and distribution of TFAP2C in HUVECs treated with different concentrations of propofol (0–50 µM) for 24 h. **D** RT-qPCR was performed to detect TFAP2C mRNA expression after treating HUVECs with different concentrations of propofol (0–50 µM) for 24 h. **E** The expression of TFAP2C in HUVECs after 100 µg/ml CHX treatment was assessed by Western blot. **F** Quantification of TFAP2C protein degradation according to (**E**). **G** Western blot with anti-puromycin antibody and ponceau staining on HUVECs treated with 50 µM propofol for 24 h and 10 µM puromycin for 1 h. **H** Quantification of puromycin labeled peptides according to (**G**). **I** Representative photomicrographs showing the expression and distribution of puromycin labeled peptides in HUVECs treated with 50 µM propofol for 24 h and 10 µM puromycin for 1 h. All data are presented as the mean ± SD from 4–6 independent experiments. *P < 0.05, **P < 0.01

We examined alterations in TFAP2C levels in HUVECs treated with propofol. The results showed that treatment with 10–50 µM propofol for 24 h significantly inhibited TFAP2C protein expression levels in a concentration dependent manner compared with the control group. However, 5 µM propofol did not significantly affect TFAP2C protein expression levels (Fig. 5A, B). Immunofluorescence results showed that propofol inhibited the nuclear expression of TFAP2C in a concentration-dependent manner (Fig. 5C). Interestingly, the expression level of TFAP2C mRNA in HUVECs did not decrease with the increase of propofol concentration (Fig. 5D). These data suggest that propofol does not regulate TFAP2C at the transcriptional or post-transcriptional level. Next, we added 100 µg/ml CHX to HUVECs and examined the effect of propofol on the rate of TFAP2C protein degradation. The results indicated that propofol does not significantly impact the degradation rate of the TFAP2C protein (Fig. 5E**–**F). Previous studies have shown that although protein levels are generally correlated with mRNA levels, quantitative studies have demonstrated that translation and protein degradation have a major impact on protein expression control [59]. Our results show that propofol neither affects the transcriptional or post-transcriptional regulation of TFAP2C nor its protein stability. Thus, these findings suggest that propofol regulates TFAP2C at the translational level. To investigate the effect of propofol on TFAP2C translation, we first performed a puromycin intake assay. We found a decrease in TFAP2C translation levels in propofol-treated HUVECs, as indicated by a decrease in the incorporation of protein peptides (Fig. 5G–H). The same results were obtained by immunofluorescence assay (Fig. 5I).

### TFAP2C overexpression reverses the anti-angiogenic effects of Propofol in vitro

**Fig. 6:**
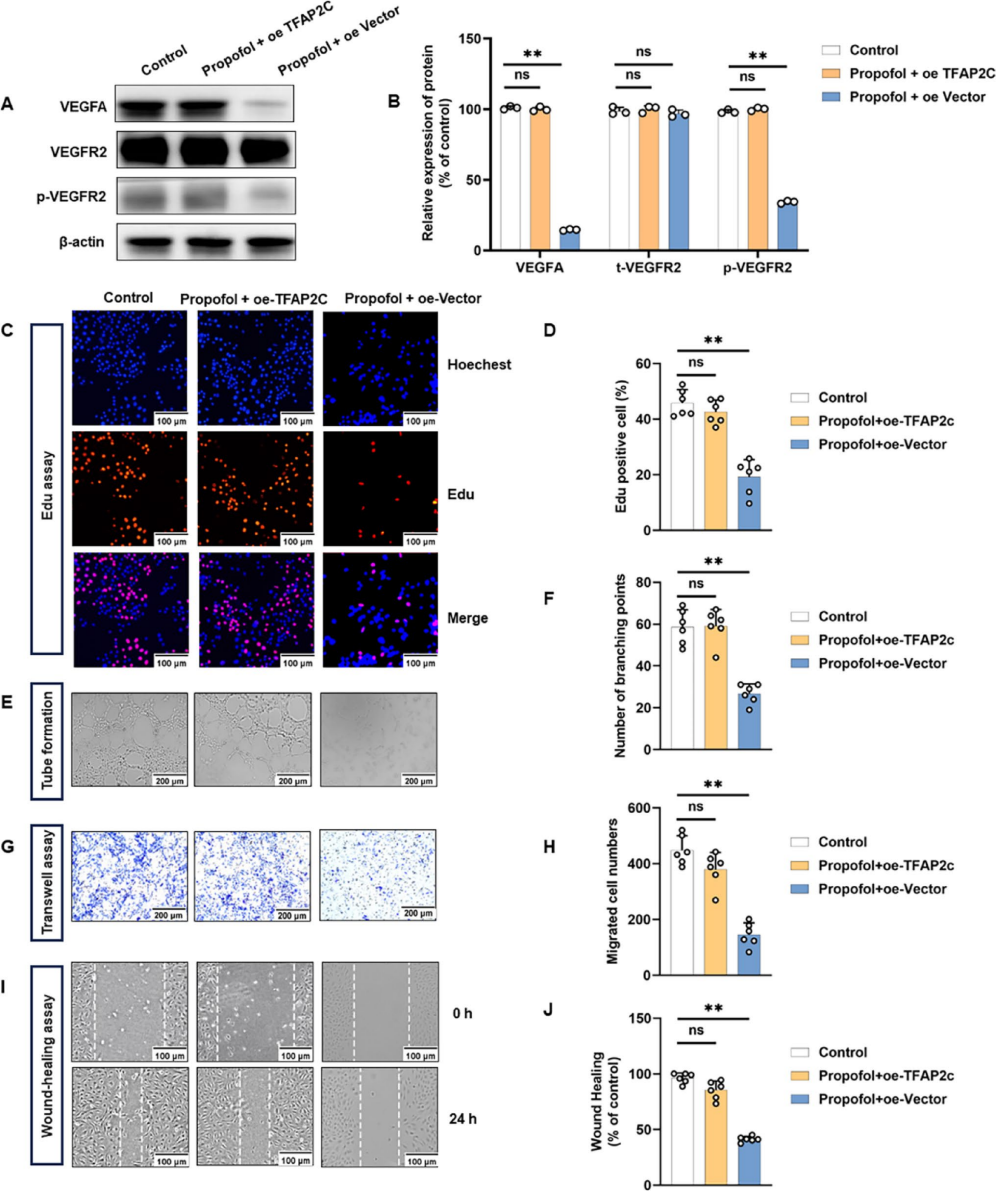
TFAP2C overexpression rescues propofol-induced inhibition of angiogenesis in vitro. HUVECs transfected with oe-Vector or overexpressing TFAP2C were treated with propofol for 24 h, with untreated cells serving as the control. **A**,** B** Western blot was performed to detect VEGFA/VEGFR2 pathway expression in different groups. **C**,** D** Representative EdU proliferation assay images of HUVECs (**C**) and quantification (**D**). **E**,** F** Representative tube-formation images of in vitro angiogenesis (E) and quantification of the branch points (**F**). **G**,** H** Representative transwell migration images of HUVECs (**G**) and quantification (**H**). **I**,** J** Representative wound healing migration images of HUVECs (**I**) and quantification (**J**). All data are presented as the mean ± SD from 3–6 independent experiments. *P < 0.05, **P < 0.01

To further verify whether TFAP2C could counteract the anti-angiogenic effect of propofol, we established TFAP2C-overexpressing HUVECs and exposed them to propofol. Western blot analysis showed that overexpression of TFAP2C significantly restored the propofol-induced reduction of VEGFA/VEGFR2 pathway (Fig. 6A, B). Functionally, EdU assays revealed that TFAP2C overexpression rescued the inhibitory effect of propofol on HUVECs proliferation (Fig. 6C, D). Consistently, tube-formation assays demonstrated that TFAP2C overexpression markedly restored tube-forming ability, as evidenced by increased branch points compared with the propofol + oeVector group (Fig. 6E, F). Similarly, transwell and wound-healing assays showed that TFAP2C overexpression reversed the suppression of migration induced by propofol (Fig. 6G–J). These findings indicate that TFAP2C overexpression rescues the propofol-induced inhibition of proliferation, migration, and tube-formation in HUVECs.

### Propofol suppresses TFAP2C protein expression through PERK/eIF2α-dependent ER stress

Propofol has been reported to suppress the mTOR/eIF4E signaling cascade [29]. To determine whether mTOR is involved in the observed translational repression of TFAP2C, we treated HUVECs with the mTOR inhibitor rapamycin and the activator MHY1485. Neither mTOR inhibition nor activation affected TFAP2C protein expression, suggesting that the mTOR pathway is not implicated in this process (Fig. S3G).

**Fig. 7:**
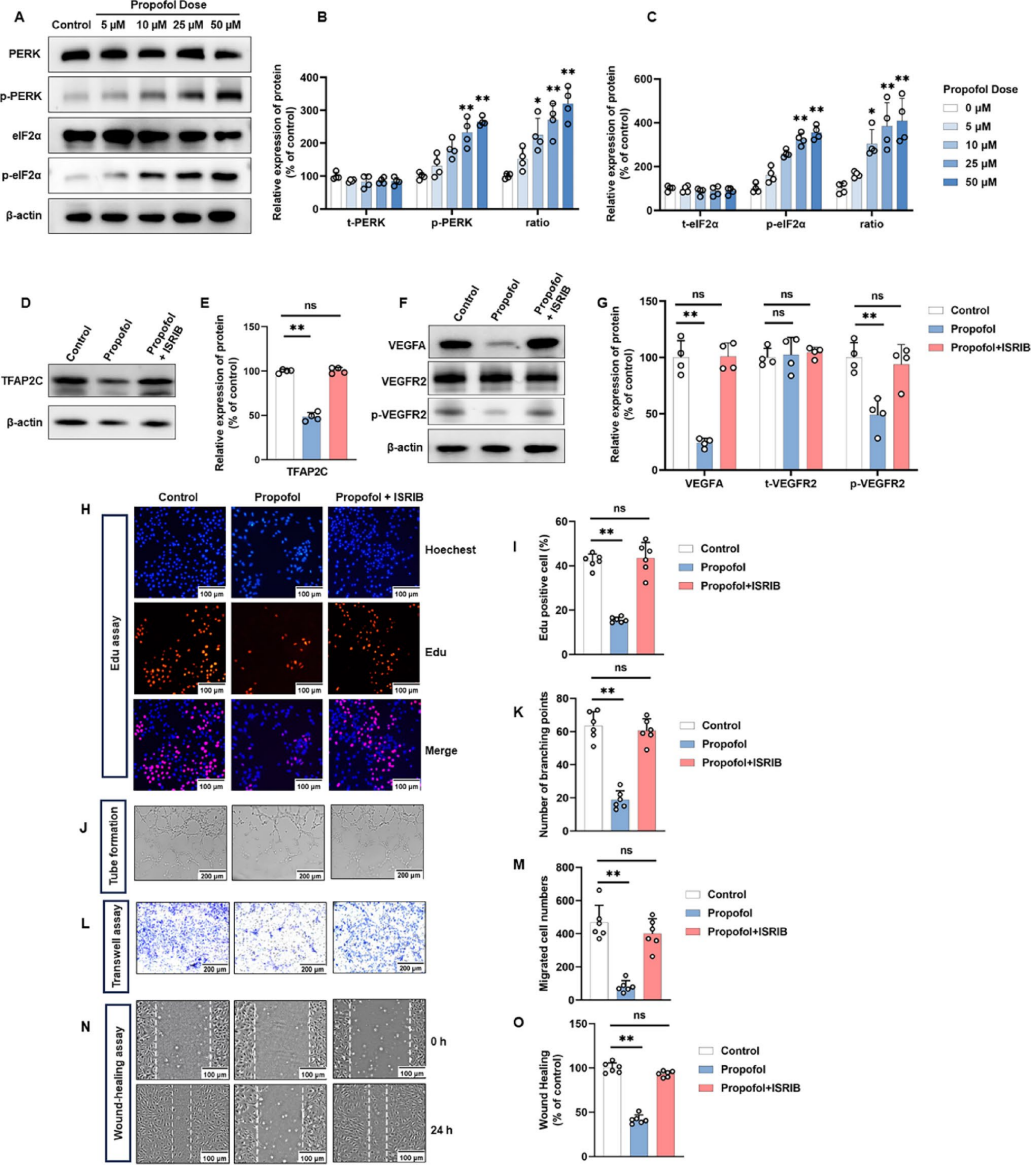
Propofol inhibits angiogenesis via ER stress–associated PERK/eIF2α signaling in HUVECs. HUVECs were treated with propofol or PERK/eIF2α signaling pathway inhibitors (ISRIB) for 24 h, with untreated cells serving as the control. **A** Western blot was performed to detect protein expression after treating HUVECs with different concentrations of propofol (0–50 µM) for 24 h. **B** Densitometric ratios for t-PERK and p-PERK expression in (**A**) were quantified. **C** Densitometric ratios for t-eIF2α and p-eIF2α expression in (**A**) were quantified. **D**,** E** Western blot was performed to detect TFAP2C protein expression after treating HUVECs with different concentrations of PERK/eIF2α signaling pathway inhibitors for 24 h. **F**,** G** Western blot was performed to detect VEGFA/VEGFR2 signaling pathway protein expression after treating HUVECs with PERK/eIF2α signaling pathway inhibitors for 24 h. **H**,** I** Representative EdU proliferation assay images of HUVECs (**H**) and quantification (**I**). **J**,** K** Representative tube-formation images of in vitro angiogenesis (**J**) and quantification of the branch points (**K**). **L**,** M** Representative transwell migration images of HUVECs (**L**) and quantification (**M**). **N**,** O** Representative wound healing migration images of HUVECs (**N**) and quantification (**O**). All data are presented as the mean ± SD from 4–6 independent experiments. *P < 0.05, **P < 0.01

Previous studies have found that propofol is associated with the activation of ERS [30–33]. The PERK arm of the unfolded protein response (UPR) is a crucial signaling pathway for cellular RES. PERK-mediated phosphorylation of eIF2α employs the upstream open reading frame (the 5’ untranslated region of the mRNA) in the regulation of translation [60]. To investigate whether the PERK/eIF2α pathway is involved in the translational regulation of TFAP2C by propofol in HUVECs, we treated HUVECs with 5–50 µM propofol and assessed changes in PERK/eIF2α and its phosphorylation levels using western blot experiments. It was found that 10–50 µM propofol significantly increased the expression levels of phosphorylated PERK and eIF2α in total proteins, implying the activation of the PERK/eIF2α pathway. While 5 µM propofol did not significantly affect the percentage of p-PERK and p-eIF2α in total protein (Fig. 7A–C).

Given that transcription factor ATF4 is a canonical downstream effector of the PERK/eIF2α pathway and has been reported to transcriptionally upregulate VEGFA [61], we next examined whether ATF4 is involved in the anti-angiogenic effect of propofol. Interestingly, in our experimental system, propofol treatment significantly activated PERK and eIF2α phosphorylation but did not lead to marked changes in ATF4 protein levels (Fig. S3H).

ISRIB (trans-isomer) is a potent PERK inhibitor that effectively reverses the effects of eIF2α phosphorylation [62]. To further demonstrate that the PERK/eIF2α pathway lies upstream of TFAP2C/VEGFA, we performed rescue experiments using ISRIB. We found that 50 µM propofol inhibited TFAP2C protein expression compared with the control group, whereas inhibition of the PERK/eIF2α pathway reversed these effects (Fig. 7D, E). Furthermore, 50 µM propofol suppressed VEGFA protein expression and inhibited VEGFR2 phosphorylation levels compared with the control group, whereas inhibition of the PERK/eIF2α pathway reversed these effects (Fig. 7F, G).

To determine whether the anti-angiogenic effects of propofol were mediated by ER stress–associated PERK/eIF2α signaling in vitro, HUVECs were treated with propofol in the presence or absence of PERK/eIF2α pathway inhibitors. EdU assays demonstrated that inhibition of PERK/eIF2α signaling partially rescued the propofol-induced suppression of HUVECs proliferation (Fig. 7H, I). Consistently, tube-formation assays showed that blocking PERK/eIF2α signaling restored tube-forming capacity, as indicated by an increased number of branch points compared with cells treated with propofol alone (Fig. 7J, K). Transwell and wound-healing assays further revealed that PERK/eIF2α inhibition mitigated the propofol-induced reduction in cell migration (Fig. 7L–O). These results indicate that propofol suppresses HUVECs proliferation, migration, and tube-formation through activation of ER stress–associated PERK/eIF2α signaling.

### Propofol suppresses angiogenesis through the PERK/eIF2α-TFAP2C-VEGFA axis in vivo

**Fig. 8:**
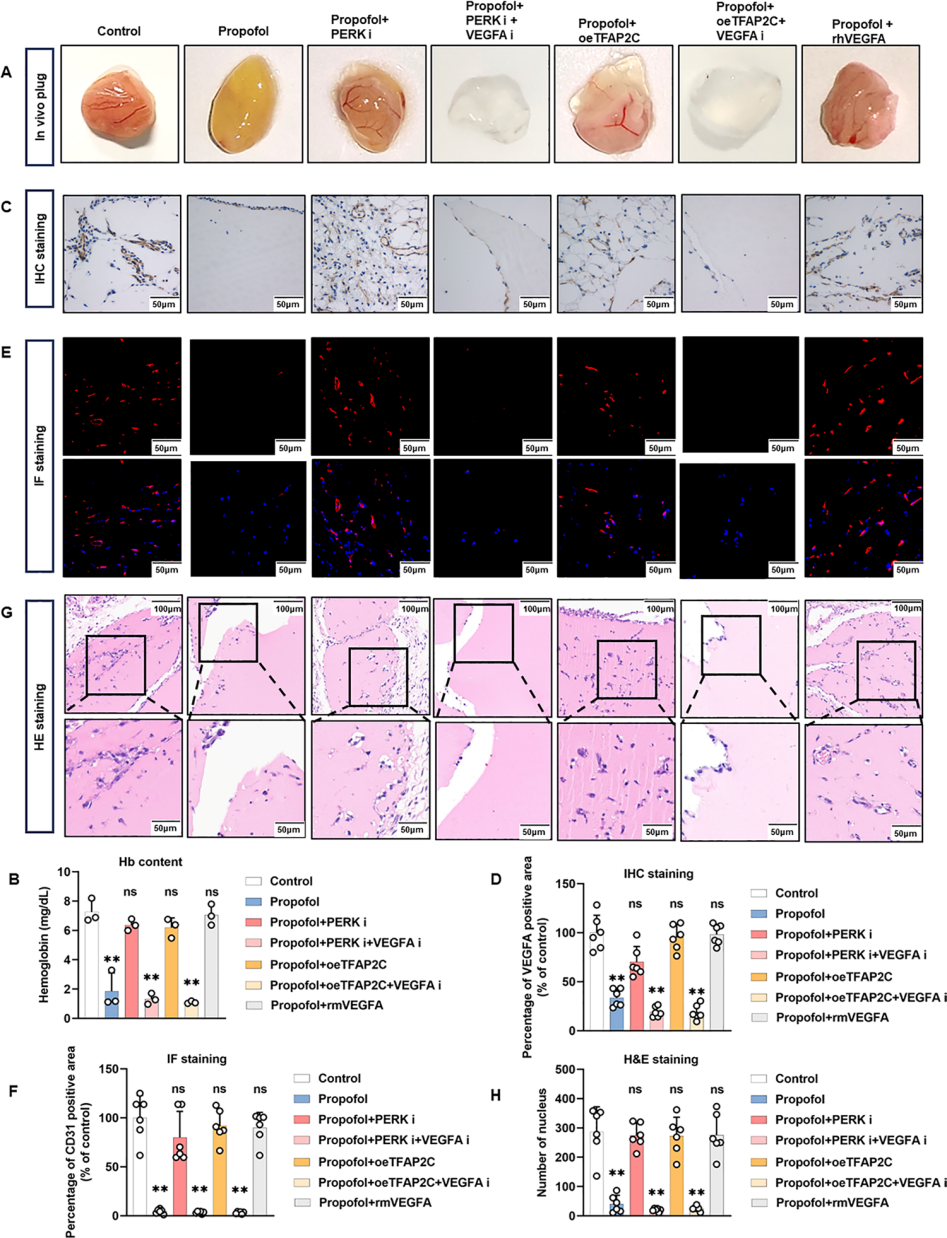
Propofol inhibits angiogenesis via PERK/eIF2α-TFAP2C-VEGFA pathway in vivo. Six-week-old female C57BL/6 mice were randomly divided into control group, propofol group, propofol + ISRIB group, propofol + ISRIB + Bevacizumab group, propofol + oeTFAP2C group, propofol + oeTFAP2C + Bevacizumab group and propofol + rmVEGFA group. Matrigel plugs were collected 7 days after subcutaneous injection. **A** Representative image of the Matrigel plugs formed after 7 days of subcutaneous injection in 6-week-old C57BL/B6 female mice. **B** The Hb concentration of the Matrigel plugs were calculated. **C**,** D** Representative images of IHC staining for VEGFA (**C**) with quantitative analysis of vessel density using VEGFA positive area (**D**). **E**,** F** Representative images of IF staining for CD31 (**E**) with quantitative analysis of vessel density using CD31 positive area (**F**). **G**,** H** Representative photomicrographs of H&E staining from Matrigel sections (**G**) and cell counts under each field of view in H&E staining (H). All data are presented as the mean ± SD from 3–6 independent experiments. *P < 0.05, **P < 0.01

To evaluate the anti-angiogenic effect of propofol in vivo, Matrigel plug assays were performed. One week after subcutaneous implantation, plugs from propofol-treated mice showed markedly reduced hemoglobin content compared with controls, indicating suppressed angiogenesis (Fig. 8A, B). To test whether this effect involves the PERK/eIF2α–TFAP2C–VEGFA axis, rescue experiments were performed.

Pharmacological inhibition of PERK signaling or overexpression of TFAP2C partially restored hemoglobin accumulation, demonstrating that propofol-mediated angiogenesis inhibition depends on this pathway. Similarly, co-administration of rmVEGFA also rescued hemoglobin content, confirming that VEGFA is a key downstream effector (Fig. 8A, B). Importantly, VEGFA neutralization with bevacizumab abolished the rescue induced by ISRIB or TFAP2C overexpression, indicating that the anti-angiogenic effects of PERK/eIF2α–TFAP2C modulation are mediated by VEGFA.

Consistent with hemoglobin measurements, both VEGFA immunohistochemistry and CD31 immunofluorescence showed that microvessel density was significantly reduced in propofol-treated plugs. This reduction was partially rescued by ISRIB, TFAP2C overexpression, or rmVEGFA, but remained low when bevacizumab was co-administered (Fig. 8C–F). H&E staining similarly confirmed these findings (Fig. 8G–H). Together, these results demonstrate that propofol suppresses angiogenesis in vivo through the PERK/eIF2α–TFAP2C–VEGFA pathway, and that restoration of VEGFA activity is sufficient to rescue the anti-angiogenic effect.

## Discussion

In this study, we provide compelling evidence that propofol directly inhibits angiogenesis in ECs through the PERK/eIF2α–TFAP2C–VEGFA/VEGFR2 signaling axis. While previous studies predominantly focused on propofol’s effects on tumor angiogenesis by modulating VEGF secretion from cancer cells, our findings demonstrate that propofol also acts directly on ECs in non-tumor contexts, expanding its pharmacological significance beyond anesthesia and tumor biology.

Propofol (2,6-dipropylphenol), a fat-soluble intravenous drug with fast onset of action, rapid recovery, and few side effects, is therefore commonly used for surgical anesthesia or intensive care [63]. Beyond its anesthetic role, propofol has been shown to modulate the inflammatory response, oxidative stress, and barrier function of ECs [20–25]. In addition, propofol has been reported to modulate VEGF expression in tumor cells and influence their interactions with ECs, thereby disrupting the tumor microenvironment [26–29]. For example, Wang et al. demonstrated that propofol suppressed VEGF/VEGFR2 signaling and affected the mTOR/eIF4E pathway in tumor–endothelial co-culture models [29]. Nevertheless, its direct effects on ECs–mediated angiogenesis in non-tumor pathological contexts remain largely unexplored. Clinically, plasma concentrations of propofol during general anaesthesia typically range from 2 to 6 µg/mL [64–68]. We selected a concentration range of 5–50 µM to encompass clinically relevant levels while permitting assessment of potential dose-dependent effects [18, 29, 69–71]. Although this range approximates the clinically achievable plasma concentrations, the in vitro setting does not account for pharmacokinetic factors such as protein binding, metabolism, and tissue partitioning, which may affect the actual bioavailable concentration in vivo.

In the current study, we found that 10, 25, and 50 µM propofol inhibited the proliferation, migration, and tube formation ability of HUVECs in a concentration-dependent manner. In contrast, propofol at 5 µM had no significant effect on the angiogenesis of HUVECs in vitro. Notably, the reduction in cell proliferation induced by propofol reflected growth arrest rather than cytotoxicity. Importantly, our in vivo Matrigel plug assay confirmed that propofol effectively suppresses angiogenesis, indicating that its anti-angiogenic effects are not limited to in vitro conditions. These findings highlight a broader role of propofol in modulating vascular remodeling under pathological conditions characterized by aberrant angiogenesis.

ECs are essential regulators of angiogenesis, and their proliferation, migration, and tube formation are tightly controlled by VEGFA/VEGFR2 signaling [15, 66]. VEGFA plays an important role in several physiological angiogenesis processes, such as the female physiological cycle and wound healing [67, 68]. Its aberrant expression or activity is closely associated with the development of various pathological conditions, such as tumors, diabetic retinopathy, and atherosclerosis, etc [14, 69, 70]. The action of VEGFA is mainly achieved through the signaling pathway of its downstream receptor, VEGFR2 [14]. VEGFR2 is a receptor tyrosine kinase that is mainly expressed on the surface of ECs [71]. VEGFA binds to VEGFR2 and activates the phosphorylation of VEGFR2 and a series of downstream signaling pathways, such as PI3K/Akt, MAPK, and PLCγ, which in turn promotes ECs proliferation, migration, and tube formation [72–75]. Consistent with earlier studies showing propofol can disrupt tumor angiogenesis, we observed that propofol treatment significantly reduced VEGFA expression and impaired VEGFR2 activation in ECs.

Given the consistent changes in mRNA and protein levels of VEGFA, we hypothesized that propofol might inhibit the transcriptional levels of VEGFA. We identified TFAP2C as a potential transcription factor related to VEGFA by public database prediction. Although the transcriptional effects of TFAP2C on VEGFA have not been reported, Ren et al. found that TFAP2C reversed ECs injury by promoting KLF10 transcription [44]. This implies that TFAP2C may have a potential protective effect on ECs. To confirm our conjecture, we knocked down TFAP2C in HUVECs and found that the proliferation, migration, and tube formation of HUVECs were attenuated. Meanwhile, knockdown of TFAP2C inhibited activation of the VEGFA/VEGFR2 signaling axis. To explore whether TFAP2C directly regulates the transcriptional activity of VEGFA in pathologic angiogenesis, we performed luciferase reporter gene and CHIP experiments. Our results revealed a direct binding of TFAP2C in the VEGFA promoter region and the binding portion is located at 1149–1163 bp of the VEGFA promoter sequence. These findings indicate that TFAP2C functions as a novel transcriptional activator of VEGFA in endothelial cells. Furthermore, our data suggest that modulation of TFAP2C activity could represent a therapeutic strategy for controlling abnormal angiogenesis in disease contexts beyond tumors, such as diabetic vascular complications or ischemic disorders.

Previous studies have found that propofol is associated with activation of the ERS signaling pathway, which refers to the accumulation of misfolded or unfolded proteins in the endoplasmic reticulum when cells are exposed to noxious stimuli or physiological changes [76]. This process triggers a series of downstream signaling cascades, such as activation of PERK/eIF2α signaling pathway [36, 37]. The eIF protein family is a group of key factors involved in the translation initiation process of eukaryotic proteins [35, 77–80]. eIF2α, a member of the eIF protein family, is also involved in the regulation of translation [77]. Yang et al. found that African swine fever virus RNA polymerase subunits C315R and H359L inhibit host translation by activating the PERK-eIF2α pathway [81]. In contrast, Wang et al. found that ADAR1 deaminase facilitated the translation process by reducing PKR-dependent eIF2α phosphorylation [82]. Previous studies have established that PERK activation during ER stress primarily regulates the classical downstream transcription factor ATF4, which mediates a variety of cellular responses to stress [83]. Interestingly, in our experimental system, although propofol robustly induces eIF2α phosphorylation, ATF4 protein levels remain unchanged, likely because ATF4 translation depends on both sufficient mRNA availability and uORF-mediated reinitiation, which can be restricted under specific stress conditions [84–88].

Instead, we identified TFAP2C as a novel downstream effector of PERK in ECs. Propofol-induced PERK activation suppressed TFAP2C translation, leading to decreased VEGFA expression and impaired VEGFR2 signaling. These findings indicate that, beyond the canonical PERK-eIF2α-ATF4 axis, alternative transcription factors such as TFAP2C may mediate the effects of ER stress on angiogenesis, revealing a previously unrecognized mechanism linking ER stress to endothelial function.

In summary, our study demonstrates that propofol directly inhibits ECs–mediated angiogenesis through the PERK/eIF2α–TFAP2C–VEGFA/VEGFR2 signaling axis. While previous studies primarily focused on the role of propofol in tumor-associated angiogenesis via modulation of VEGF secretion from cancer cells, our findings reveal that propofol acts directly on ECs in non-tumor pathological contexts, highlighting its broader pharmacological significance. Notably, propofol-induced activation of the PERK/eIF2α pathway suppresses TFAP2C translation without affecting its mRNA levels, linking ER stress to translational control of a key transcription factor. Unlike the classical PERK/eIF2α/ATF4 axis, ATF4 was not involved in this regulatory process in our system, suggesting that alternative transcription factors such as TFAP2C can serve as novel effectors of ER stress in endothelial cells. Beyond these initial findings, the identification of TFAP2C as a novel transcription factor for VEGFA opens new avenues for understanding the regulation of pathological angiogenesis. Future studies should evaluate the therapeutic potential of targeting TFAP2C in diverse disease settings characterized by abnormal vascular growth.

## Supplementary Information

Below is the link to the electronic supplementary material.

Supplementary Material 1

Supplementary Material 2

## Data Availability

Data will be made available on request.

## Abbreviations

ECs: Endothelial cells
VEGF: Vascular endothelial growth factor
rhVEGFA: Recombinant human VEGFA
rmVEGFA: Recombinant mouse VEGFA
rhFGF2: Recombinant human FGF2
ERS: Endoplasmic reticulum stress
PERK: Protein kinase R (PKR)-like endoplasmic reticulum kinase
eIF2α: Eukaryotic initiation factor 2α
UPR: unfolded protein response
HUVECs: Human umbilical vein endothelial cells
KLF10K: rüppel-like factor 10
HEK 293T: Human embryonic kidney 293T
SD: Standard deviation
qRT‒PCR: RNA isolation and quantitative real‑time PCR
WB: Western blot
IF: Immunofluorescence
ChIP: Chromatin immunoprecipitation
CHX: Cycloheximide
IHC: Immunohistochemical
H&E: Hematoxylin & Eosin
DMSO: Dimethyl sulfoxide
EMT: Epithelial-mesenchymal transition
TSS: Transcription start site
Hb: Hemoglobin

## References

[1] Feng Q, Yu C, Guo L et al (2025) DCBLD1 modulates angiogenesis by regulation of the VEGFR-2 endocytosis in endothelial cells. Arterioscler Thromb Vasc Biol 45:198–217. 10.1161/ATVBAHA.123.320443 39665138 10.1161/ATVBAHA.123.320443

[2] Ribatti D, Crivellato E (2012) Sprouting angiogenesis, a reappraisal. Dev Biol 372:157–165. 10.1016/j.ydbio.2012.09.018 23031691 10.1016/j.ydbio.2012.09.018

[3] Wang Y, Niu C, Yu G et al (2025) NIR-responsive injectable nanocomposite hydrogels with enhanced angiogenesis for promoting full-thickness wound healing. Int J Biol Macromol 288:138688. 10.1016/j.ijbiomac.2024.138688 39672424 10.1016/j.ijbiomac.2024.138688

[4] Ning W, Yang J, Ni R et al (2025) Hypoxia induced cellular and Exosomal RPPH1 promotes breast cancer angiogenesis and metastasis through stabilizing the IGF2BP2/FGFR2 axis. Oncogene 44:147–164. 10.1038/s41388-024-03213-y 39496940 10.1038/s41388-024-03213-y

[5] Chen Y, Dong J, Liu W et al (2024) Polysaccharides from ostrea rivularis alleviate type II diabetes induced-retinopathy and VGEF165-induced angiogenesis via PI3K/AKT signaling pathway. Int J Biol Macromol 279:135547. 10.1016/j.ijbiomac.2024.135547 39265902 10.1016/j.ijbiomac.2024.135547

[6] Wei Y, Li Y, Shu Y et al (2025) The new anti-angiogenesis perspective of rheumatoid arthritis with geniposide: reducing the extracellular release of HSP70 in HUVECs. Int Immunopharmacol 144:113645. 10.1016/j.intimp.2024.113645 39571270 10.1016/j.intimp.2024.113645

[7] Li Y, Zhang L, Yang W et al (2024) Notoginsenoside R1 decreases intraplaque neovascularization by governing pericyte-endothelial cell communication via Ang1/Tie2 axis in atherosclerosis. Phytother Res 38:4036–4052. 10.1002/ptr.8257 38886264 10.1002/ptr.8257

[8] Xie P, Guo L, Yu Q et al (2025) ACE2 enhances sensitivity to PD-L1 Blockade by inhibiting Macrophage-Induced immunosuppression and angiogenesis. Cancer Res 85:299–313. 10.1158/0008-5472.CAN-24-0954 39495239 10.1158/0008-5472.CAN-24-0954

[9] Grothey A, Galanis E (2009) Targeting angiogenesis: progress with anti-VEGF treatment with large molecules. Nat Rev Clin Oncol 6:507–518. 10.1038/nrclinonc.2009.110 19636328 10.1038/nrclinonc.2009.110

[10] Zhang H, Zhou J, Li J et al (2023) N6-Methyladenosine promotes translation of VEGFA to accelerate angiogenesis in lung cancer. Cancer Res 83:2208–2225. 10.1158/0008-5472.CAN-22-2449 37103476 10.1158/0008-5472.CAN-22-2449

[11] He L, Feng A, Guo H et al (2022) LRG1 mediated by ATF3 promotes growth and angiogenesis of gastric cancer by regulating the SRC/STAT3/VEGFA pathway. Gastric Cancer 25:527–541. 10.1007/s10120-022-01279-9 35094168 10.1007/s10120-022-01279-9

[12] Wu R, Zhang Y, Xu X et al (2023) Exosomal B7-H3 facilitates colorectal cancer angiogenesis and metastasis through AKT1/mTOR/VEGFA pathway. Cell Signal 109:110737. 10.1016/j.cellsig.2023.110737 37263461 10.1016/j.cellsig.2023.110737

[13] Wang H, Chen J, Chen X et al (2024) Cancer-Associated fibroblasts expressing sulfatase 1 facilitate VEGFA-Dependent microenvironmental remodeling to support colorectal cancer. Cancer Res 84:3371–3387. 10.1158/0008-5472.CAN-23-3987 39250301 10.1158/0008-5472.CAN-23-3987

[14] Chen L, Xie X, Wang T et al (2023) ARL13B promotes angiogenesis and glioma growth by activating VEGFA-VEGFR2 signaling. Neuro Oncol 25:871–885. 10.1093/neuonc/noac245 36322624 10.1093/neuonc/noac245 PMC10158193

[15] Zhang Q, Lu S, Li T et al (2019) ACE2 inhibits breast cancer angiogenesis via suppressing the VEGFa/VEGFR2/ERK pathway. J Exp Clin Cancer Res 38:173. 10.1186/s13046-019-1156-5 31023337 10.1186/s13046-019-1156-5 PMC6482513

[16] Wen J, Xue L, Wei Y et al (2024) YTHDF2 is a therapeutic target for HCC by suppressing immune evasion and angiogenesis through ETV5/PD-L1/VEGFA axis. Adv Sci (Weinh) 11:e2307242. 10.1002/advs.202307242 38247171 10.1002/advs.202307242 PMC10987122

[17] Lan L, Liao J, Qin L et al (2025) The effects of Ciprofol on haemodynamics under general anaesthesia during thoracoscopic surgery: a randomised, double-blind, controlled trial. BMC Anesthesiol 25:168. 10.1186/s12871-025-03054-6 40211149 10.1186/s12871-025-03054-6 PMC11984254

[18] Ye T, Fan Y, Zeng X et al (2025) Induction of M1 polarization in BV2 cells by Propofol intervention promotes perioperative neurocognitive disorders through the NGF/CREB signaling pathway: an experimental research. Int J Surg. 10.1097/JS9.0000000000002257 39869375 10.1097/JS9.0000000000002257 PMC12372762

[19] Zhang X, Wei K-Y, Huang D (2024) Effect of Propofol in the cardiovascular system and its related mechanism research progress. Niger J Clin Pract 27:938–944. 10.4103/njcp.njcp_292_24 39212428 10.4103/njcp.njcp_292_24

[20] Ding X-W, Sun X, Shen X-F et al (2019) Propofol attenuates TNF-α-induced MMP-9 expression in human cerebral microvascular endothelial cells by inhibiting Ca2+/CAMK II/ERK/NF-κB signaling pathway. Acta Pharmacol Sin 40:1303–1313. 10.1038/s41401-019-0258-0 31235816 10.1038/s41401-019-0258-0 PMC6786358

[21] Sun Z, Lv J, Zhu Y et al (2015) Desflurane preconditioning protects human umbilical vein endothelial cells against anoxia/reoxygenation by upregulating NLRP12 and inhibiting non-canonical nuclear factor-κB signaling. Int J Mol Med 36:1327–1334. 10.3892/ijmm.2015.2335 26329693 10.3892/ijmm.2015.2335

[22] Zhu M, Ding J, Jiang H et al (2015) Propofol ameliorates endothelial inflammation induced by hypoxia/reoxygenation in human umbilical vein endothelial cells: role of phosphatase A2. Vascul Pharmacol 73:149–157. 10.1016/j.vph.2015.06.002 26070526 10.1016/j.vph.2015.06.002

[23] Qi J, Wu Q, Zhu X et al (2019) Propofol attenuates the adhesion of tumor and endothelial cells through inhibiting Glycolysis in human umbilical vein endothelial cells. Acta Biochim Biophys Sin (Shanghai) 51:1114–1122. 10.1093/abbs/gmz105 31650167 10.1093/abbs/gmz105

[24] Wu Q, Zhao Y, Duan W et al (2017) Propofol inhibits high glucose-induced PP2A expression in human umbilical vein endothelial cells. Vascul Pharmacol 91:18–25. 10.1016/j.vph.2017.02.002 28188886 10.1016/j.vph.2017.02.002

[25] Zhu M, Chen J, Wen M et al (2014) Propofol protects against angiotensin II-induced mouse hippocampal HT22 cells apoptosis via Inhibition of p66Shc mitochondrial translocation. Neuromolecular Med 16:772–781. 10.1007/s12017-014-8326-6 25151272 10.1007/s12017-014-8326-6

[26] Dong H, Zhou W, Han L, Zhao Q (2024) Propofol inhibits the proliferation, invasion, migration, and angiogenesis of oral squamous cell carcinoma through circ_0008898-mediated pathway. Chem Biol Drug Des 103:e14393. 10.1111/cbdd.14393 37955304 10.1111/cbdd.14393

[27] Xu Y-B, Du Q-H, Zhang M-Y et al (2013) Propofol suppresses proliferation, invasion and angiogenesis by down-regulating ERK-VEGF/MMP-9 signaling in Eca-109 esophageal squamous cell carcinoma cells. Eur Rev Med Pharmacol Sci 17:2486–2494 24089228

[28] Guo X-G, Wang S, Xu Y-B, Zhuang J (2015) Propofol suppresses invasion, angiogenesis and survival of EC-1 cells in vitro by regulation of S100A4 expression. Eur Rev Med Pharmacol Sci 19:4858–4865 26744878

[29] Wang Z, Cao B, Ji P, Yao F (2021) Propofol inhibits tumor angiogenesis through targeting VEGF/VEGFR and mTOR/eIF4E signaling. Biochem Biophys Res Commun 555:13–18. 10.1016/j.bbrc.2021.03.094 33812053 10.1016/j.bbrc.2021.03.094

[30] Kang F-C, Wang S-C, So EC et al (2019) Propofol May increase caspase and MAPK pathways, and suppress the Akt pathway to induce apoptosis in MA–10 mouse Leydig tumor cells. Oncol Rep 41:3565–3574. 10.3892/or.2019.7129 31002349 10.3892/or.2019.7129

[31] Chen X, Li K, Zhao G (2018) Propofol inhibits HeLa cells by impairing autophagic flux via AMP-Activated protein kinase (AMPK) activation and Endoplasmic reticulum stress regulated by calcium. Med Sci Monit 24:2339–2349. 10.12659/msm.909144 29667627 10.12659/MSM.909144 PMC5926273

[32] Chen X, Li L-Y, Jiang J-L et al (2018) Propofol elicits autophagy via Endoplasmic reticulum stress and calcium exchange in C2C12 myoblast cell line. PLoS ONE 13:e0197934. 10.1371/journal.pone.0197934 29795639 10.1371/journal.pone.0197934 PMC5967754

[33] Oh C-S, Hong SW, Park S et al (2022) Effect of equipotent doses of Propofol and Sevoflurane on Endoplasmic reticulum stress during breast cancer surgery. Korean J Anesthesiol 75:487–495. 10.4097/kja.21569 35760393 10.4097/kja.21569 PMC9726458

[34] Jiang M, Li X, Zhang J et al (2021) Dual Inhibition of Endoplasmic reticulum stress and oxidation stress manipulates the polarization of macrophages under hypoxia to sensitize immunotherapy. ACS Nano 15:14522–14534. 10.1021/acsnano.1c04068 34414762 10.1021/acsnano.1c04068

[35] Li H, Pan W, Li C et al (2024) Heat stress induces calcium dyshomeostasis to subsequent cognitive impairment through ERS-mediated apoptosis via SERCA/PERK/eIF2α pathway. Cell Death Discov 10:280. 10.1038/s41420-024-02047-7 38862478 10.1038/s41420-024-02047-7 PMC11167007

[36] Zou W, Bai Y, Wang X et al (2017) PERK-Phosphorylated eIF2α pathway suppresses tumor metastasis through downregulating expression of programmed death ligand 1 and CXCL5 in Triple-Negative breast cancer. Cancer Biotherapy Radiopharmaceuticals 32:282–287. 10.1089/cbr.2017.2237 29053414 10.1089/cbr.2017.2237

[37] Qiao Q, Sun C, Han C et al (2017) Endoplasmic reticulum stress pathway PERK-eIF2α confers radioresistance in oropharyngeal carcinoma by activating NF-κB. Cancer Sci 108:1421–1431. 10.1111/cas.13260 28418119 10.1111/cas.13260 PMC5497722

[38] Williams CMJ, Scibetta AG, Friedrich JK et al (2009) AP-2gamma promotes proliferation in breast tumour cells by direct repression of the CDKN1A gene. EMBO J 28:3591–3601. 10.1038/emboj.2009.290 19798054 10.1038/emboj.2009.290 PMC2782101

[39] Li L, Zheng Y-L, Jiang C et al (2019) HN1L-mediated transcriptional axis AP-2γ/METTL13/TCF3-ZEB1 drives tumor growth and metastasis in hepatocellular carcinoma. Cell Death Differ 26:2268–2283. 10.1038/s41418-019-0301-1 30778199 10.1038/s41418-019-0301-1 PMC6889153

[40] Cui G, Gao Z, Chang S et al (2022) TRIM37 augments AP-2γ transcriptional activity and cellular localization via K63-linked ubiquitination to drive breast cancer progression. Int J Biol Sci 18:4316–4328. 10.7150/ijbs.69466 35864973 10.7150/ijbs.69466 PMC9295074

[41] Wang Y, Chen S, Jiang Q et al (2020) TFAP2C facilitates somatic cell reprogramming by inhibiting c-Myc-dependent apoptosis and promoting mesenchymal-to-epithelial transition. Cell Death Dis 11:482. 10.1038/s41419-020-2684-9 32587258 10.1038/s41419-020-2684-9 PMC7316975

[42] Zeng T-T, Deng T-H, Liu Z et al (2022) HN1L/AP-2γ/PLK1 signaling drives tumor progression and chemotherapy resistance in esophageal squamous cell carcinoma. Cell Death Dis 13:1026. 10.1038/s41419-022-05478-1 36476988 10.1038/s41419-022-05478-1 PMC9729194

[43] Chang T-H, Tsai M-F, Gow C-H et al (2017) Upregulation of microRNA-137 expression by slug promotes tumor invasion and metastasis of non-small cell lung cancer cells through suppression of TFAP2C. Cancer Lett 402:190–202. 10.1016/j.canlet.2017.06.002 28610956 10.1016/j.canlet.2017.06.002

[44] Ren Y, Shi J, Liu S et al (2023) Transcription factor AP-2 gamma/Krüppel-like factor 10 axis is involved in miR-3656-related dysfunction of endothelial cells in hypertension. J Hypertens 41:554. 10.1097/HJH.0000000000003359 36723462 10.1097/HJH.0000000000003359

[45] Zhou B, Ma R, Si W et al (2013) MicroRNA-503 targets FGF2 and VEGFA and inhibits tumor angiogenesis and growth. Cancer Lett 333:159–169. 10.1016/j.canlet.2013.01.028 23352645 10.1016/j.canlet.2013.01.028

[46] Wei H, Cao C, Wei X et al (2020) Circular RNA circvegfc accelerates high glucose-induced vascular endothelial cells apoptosis through miR-338-3p/HIF-1α/VEGFA axis. Aging 12:14365–14375. 10.18632/aging.103478 32680978 10.18632/aging.103478 PMC7425483

[47] Xiao B, Wang G, Huo H, Li W (2021) Identification of HIF-1α/VEGFA signaling pathway and transcription factors in Kashin-Beck disease by integrated bioinformatics analysis. Exp Ther Med 22:1115. 10.3892/etm.2021.10549 34504569 10.3892/etm.2021.10549 PMC8383754

[48] Fu X, Zhai S, Yuan J (2018) Interleukin-6 (IL-6) triggers the malignancy of hemangioma cells via activation of HIF-1α/VEGFA signals. Eur J Pharmacol 841:82–89. 10.1016/j.ejphar.2018.10.022 30342949 10.1016/j.ejphar.2018.10.022

[49] Yao L, Zhai W, Jiang Z et al (2024) The inhibitory effects of Propofol on colorectal cancer progression through the NF-κB/HIF-1α signaling pathway. Anticancer Agents Med Chem 24:878–888. 10.2174/0118715206283884240326170501 38571352 10.2174/0118715206283884240326170501

[50] Meng Q, Li S, Liu Y et al (2019) Circular RNA circSCAF11 accelerates the glioma tumorigenesis through the miR-421/SP1/VEGFA axis. Mol Ther Nucleic Acids 17:669–677. 10.1016/j.omtn.2019.06.022 31400609 10.1016/j.omtn.2019.06.022 PMC6700438

[51] Wang J, Zhao E, Geng B et al (2024) Downregulation of UBB potentiates SP1/VEGFA-dependent angiogenesis in clear cell renal cell carcinoma. Oncogene 43:1386–1396. 10.1038/s41388-024-03003-6 38467852 10.1038/s41388-024-03003-6 PMC11065696

[52] Donovan K, Alekseev O, Qi X et al (2014) O-GlcNAc modification of transcription factor Sp1 mediates hyperglycemia-induced VEGF-A upregulation in retinal cells. Invest Ophthalmol Vis Sci 55:7862–7873. 10.1167/iovs.14-14048 25352121 10.1167/iovs.14-14048 PMC4541483

[53] Qin L, Xu Y, Xu Y et al (2015) NCOA1 promotes angiogenesis in breast tumors by simultaneously enhancing both HIF1α- and AP-1-mediated VEGFa transcription. Oncotarget 6:23890–23904. 10.18632/oncotarget.4341 26287601 10.18632/oncotarget.4341 PMC4695159

[54] Shih SC, Claffey KP (2001) Role of AP-1 and HIF-1 transcription factors in TGF-beta activation of VEGF expression. Growth Factors 19:19–34. 10.3109/08977190109001073 11678207 10.3109/08977190109001073

[55] Guo S, Li J, Huang Z et al (2022) The CBS-H2S axis promotes liver metastasis of colon cancer by upregulating VEGF through AP-1 activation. Br J Cancer 126:1055–1066. 10.1038/s41416-021-01681-7 34952931 10.1038/s41416-021-01681-7 PMC8979992

[56] Cs M, C L et al (2022) J N, Macrophage IL-1β promotes arteriogenesis by autocrine STAT3- and NF-κB-mediated transcription of pro-angiogenic VEGF-A. 10.1016/j.celrep.2022.110309 10.1016/j.celrep.2022.110309 PMC8865931 35108537

[57] Greenberger S, Adini I, Boscolo E et al (2010) Targeting NF-κB in infantile hemangioma-derived stem cells reduces VEGF-A expression. Angiogenesis 13:327–335. 10.1007/s10456-010-9189-6 20872175 10.1007/s10456-010-9189-6 PMC3034388

[58] Trebec-Reynolds DP, Voronov I, Heersche JNM, Manolson MF (2010) VEGF-A expression in osteoclasts is regulated by NF-kappaB induction of HIF-1alpha. J Cell Biochem 110:343–351. 10.1002/jcb.22542 20432243 10.1002/jcb.22542

[59] Munro V, Kelly V, Messner CB, Kustatscher G (2024) Cellular control of protein levels: A systems biology perspective. Proteomics 24:2200220. 10.1002/pmic.202200220 10.1002/pmic.202200220 38012370

[60] Wek RC, Cavener DR (2007) Translational control and the unfolded protein response. Antioxid Redox Signal 9:2357–2371. 10.1089/ars.2007.1764 17760508 10.1089/ars.2007.1764

[61] Oskolkova OV, Afonyushkin T, Leitner A et al (2008) ATF4-dependent transcription is a key mechanism in VEGF up-regulation by oxidized phospholipids: critical role of oxidized sn-2 residues in activation of unfolded protein response. Blood 112:330–339. 10.1182/blood-2007-09-112870 18451308 10.1182/blood-2007-09-112870 PMC2442744

[62] Sidrauski C, Acosta-Alvear D, Khoutorsky A et al (2013) Pharmacological brake-release of mRNA translation enhances cognitive memory. Elife 2:e00498. 10.7554/eLife.00498 23741617 10.7554/eLife.00498 PMC3667625

[63] Sun F, He Y, Yang Z et al (2024) Propofol pretreatment inhibits ferroptosis and alleviates myocardial ischemia-reperfusion injury through the SLC16A13-AMPK-GPX4 pathway. Biomed Pharmacother 179:117345. 10.1016/j.biopha.2024.117345 39208667 10.1016/j.biopha.2024.117345

[64] Kasuya Y, Govinda R, Rauch S et al (2009) The correlation between bispectral index and observational sedation scale in volunteers sedated with Dexmedetomidine and Propofol. Anesth Analg 109:1811–1815. 10.1213/ANE.0b013e3181c04e58 19923507 10.1213/ANE.0b013e3181c04e58

[65] Fanti L, Agostoni M, Casati A et al (2004) Target-controlled Propofol infusion during monitored anesthesia in patients undergoing ERCP. Gastrointest Endosc 60:361–366. 10.1016/s0016-5107(04)01713-4 15332024 10.1016/s0016-5107(04)01713-4

[66] Tirel O, Wodey E, Harris R et al (2008) Variation of bispectral index under TIVA with Propofol in a paediatric population. Br J Anaesth 100:82–87. 10.1093/bja/aem339 18070785 10.1093/bja/aem339 PMC2657834

[67] Lan H, Cao H, Liu S et al (2024) Efficacy of remimazolam tosilate versus Propofol for total intravenous anaesthesia in urological surgery: A randomised clinical trial. Eur J Anaesthesiol 41:208–216. 10.1097/EJA.0000000000001938 38165145 10.1097/EJA.0000000000001938

[68] Kazama T, Takeuchi K, Ikeda K et al (2000) Optimal Propofol plasma concentration during upper Gastrointestinal endoscopy in young, middle-aged, and elderly patients. Anesthesiology 93:662–669. 10.1097/00000542-200009000-00014 10969298 10.1097/00000542-200009000-00014

[69] Lu Z, Shen J, Chen X et al (2022) Propofol upregulates MicroRNA-30b to inhibit excessive autophagy and apoptosis and attenuates Ischemia/Reperfusion injury in vitro and in patients. Oxid Med Cell Longev 2022:2109891. 10.1155/2022/2109891 35401922 10.1155/2022/2109891 PMC8986434

[70] Mathy-Hartert M, Deby-Dupont G, Hans P et al (1998) Protective activity of propofol, Diprivan and intralipid against active oxygen species. Mediators Inflamm 7:327–333. 10.1080/09629359890848 9883967 10.1080/09629359890848 PMC1781863

[71] Zhu M, Chen J, Jiang H, Miao C (2013) Propofol protects against high glucose-induced endothelial adhesion molecules expression in human umbilical vein endothelial cells. Cardiovasc Diabetol 12:13. 10.1186/1475-2840-12-13 23311470 10.1186/1475-2840-12-13 PMC3579710

[72] Liao Q, Shi H, Yang J et al (2024) FTO elicits tumor neovascularization in cancer-associated fibroblasts through eliminating m6A modifications of multiple pro-angiogenic factors. Cancer Lett 592:216911. 10.1016/j.canlet.2024.216911 38685450 10.1016/j.canlet.2024.216911

[73] Wang Z, Xu H, Xue B et al (2024) MSC-derived Exosomal circMYO9B accelerates diabetic wound healing by promoting angiogenesis through the hnRNPU/CBL/KDM1A/VEGFA axis. Commun Biol 7:1700. 10.1038/s42003-024-07367-z 39725699 10.1038/s42003-024-07367-z PMC11671590

[74] Malik S, Day K, Perrault I et al (2006) Reduced levels of VEGF-A and MMP-2 and MMP-9 activity and increased TNF-alpha in menstrual endometrium and effluent in women with menorrhagia. Hum Reprod 21:2158–2166. 10.1093/humrep/del089 16585124 10.1093/humrep/del089

[75] Yang Y (2025) YAP1 overexpression aggravates the progress of diabetic retinopathy by activating the TUG1/miR-144-3p/VEGFA signaling pathway in the hypoxia-induced DR MRMECs model. Tissue Cell 92:102620. 10.1016/j.tice.2024.102620 39615227 10.1016/j.tice.2024.102620

[76] Lu W, Wan G, Zhu H et al (2024) MiR-497-5p regulates ox-LDL-induced dysfunction in vascular endothelial cells by targeting VEGFA/p38/MAPK pathway in atherosclerosis. Heliyon 10:e28887. 10.1016/j.heliyon.2024.e28887 38601630 10.1016/j.heliyon.2024.e28887 PMC11004747

[77] Tao B-B, Liu S-Y, Zhang C-C et al (2013) VEGFR2 functions as an H2S-targeting receptor protein kinase with its novel Cys1045-Cys1024 disulfide bond serving as a specific molecular switch for hydrogen sulfide actions in vascular endothelial cells. Antioxid Redox Signal 19:448–464. 10.1089/ars.2012.4565 23199280 10.1089/ars.2012.4565 PMC3704125

[78] Basagiannis D, Zografou S, Murphy C et al (2016) VEGF induces signalling and angiogenesis by directing VEGFR2 internalisation through macropinocytosis. J Cell Sci 129:4091–4104. 10.1242/jcs.188219 27656109 10.1242/jcs.188219

[79] Zheng X, Liu X, Wang Z et al (2025) Selenium-Chondroitin sulfate nanoparticles inhibit angiogenesis by regulating the VEGFR2-Mediated PI3K/Akt pathway. Mar Drugs 23:22. 10.3390/md23010022 39852524 10.3390/md23010022 PMC11766607

[80] Sjöberg E, Melssen M, Richards M et al (2023) Endothelial VEGFR2-PLCγ signaling regulates vascular permeability and antitumor immunity through eNOS/Src. J Clin Invest 133:e161366. 10.1172/JCI161366 37651195 10.1172/JCI161366 PMC10575733

[81] Duan J, Hu H, Feng L et al (2017) Silica nanoparticles inhibit macrophage activity and angiogenesis via VEGFR2-mediated MAPK signaling pathway in zebrafish embryos. Chemosphere 183:483–490. 10.1016/j.chemosphere.2017.05.138 28570891 10.1016/j.chemosphere.2017.05.138

[82] Chen X, Cubillos-Ruiz JR (2021) Endoplasmic reticulum stress signals in the tumour and its microenvironment. Nat Rev Cancer 21:71–88. 10.1038/s41568-020-00312-2 33214692 10.1038/s41568-020-00312-2 PMC7927882

[83] Grove DJ, Levine DJ, Kearse MG (2023) Increased levels of eIF2A inhibit translation by sequestering 40S ribosomal subunits. Nucleic Acids Res 51:9983–10000. 10.1093/nar/gkad683 37602404 10.1093/nar/gkad683 PMC10570035

[84] Deng J, Harding HP, Raught B et al (2002) Activation of GCN2 in UV-irradiated cells inhibits translation. Curr Biol 12:1279–1286. 10.1016/s0960-9822(02)01037-0 12176355 10.1016/s0960-9822(02)01037-0

[85] Jiang H-Y, Wek RC (2005) GCN2 phosphorylation of eIF2alpha activates NF-kappaB in response to UV irradiation. Biochem J 385:371–380. 10.1042/BJ20041164 15355306 10.1042/BJ20041164 PMC1134707

[86] Dey S, Baird TD, Zhou D et al (2010) Both transcriptional regulation and translational control of ATF4 are central to the integrated stress response. J Biol Chem 285:33165–33174. 10.1074/jbc.M110.167213 20732869 10.1074/jbc.M110.167213 PMC2963398

[87] Rutkowski DT, Arnold SM, Miller CN et al (2006) Adaptation to ER stress is mediated by differential stabilities of Pro-Survival and Pro-Apoptotic mRNAs and proteins. PLoS Biol 4:e374. 10.1371/journal.pbio.0040374 17090218 10.1371/journal.pbio.0040374 PMC1634883

[88] Vattem KM, Wek RC (2004) Reinitiation involving upstream ORFs regulates ATF4 mRNA translation in mammalian cells. Proc Natl Acad Sci U S A 101:11269–11274. 10.1073/pnas.0400541101 15277680 10.1073/pnas.0400541101 PMC509193

