# Supplementary material for: Propofol attenuates angiogenesis by activating endoplasmic reticulum stress to suppress TFAP2C-driven VEGFA transcription
Source: Apoptosis. 2026 Jan 12;31(1):42. doi: 10.1007/s10495-025-02214-w (PMC12795951; doi:10.1007/s10495-025-02214-w)
Supplement: Supplementary file 1 — Supplementary Material 1 [file 10495_2025_2214_MOESM1_ESM.docx]

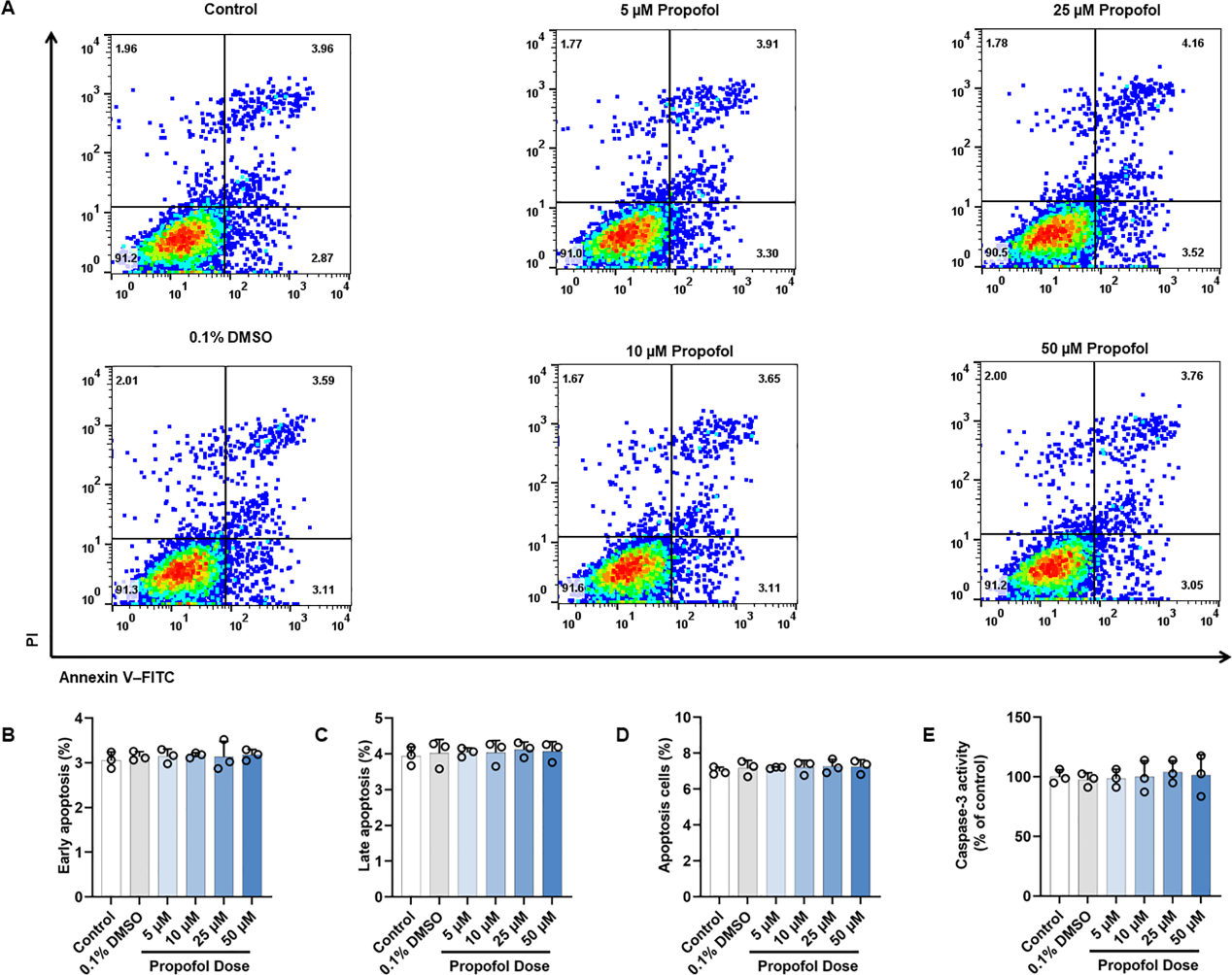


**Fig. S1 Propofol does not induce apoptosis in HUVECs.**

HUVECs cell lines were treated with different concentrations of propofol (5-50 µM), while a blank control and solvent control were set up.
**(A)** Representative flow cytometry plots showing Annexin V–FITC/PI staining of HUVECs treated with 0–50 μM propofol for 24 h.
**(B)** Quantification of early apoptotic cells.
**(C)** Quantification of late apoptotic cells.
**(D)** Quantification of total apoptotic cells.

**(E)** Quantification of relative caspase-3 activity normalized to control.
All data are presented as the mean ± SD from 3 independent experiments.


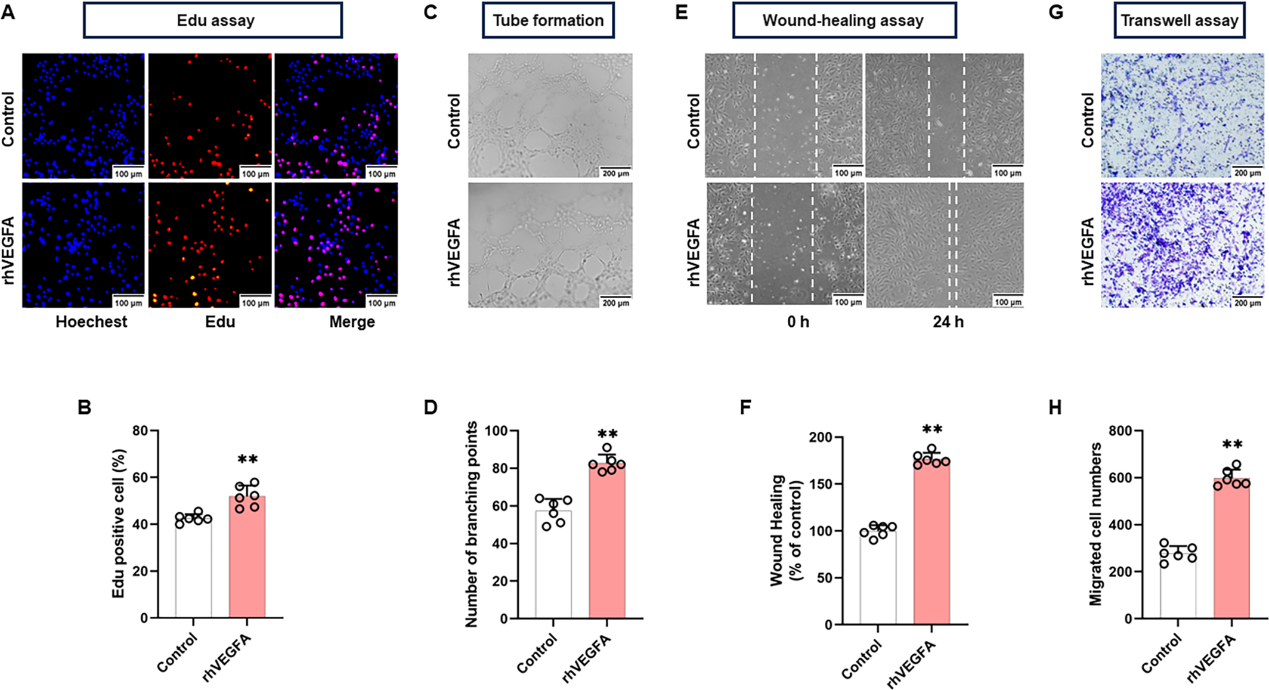


**Fig. S2 Exogenous VEGFA enhances HUVECs angiogenic behaviors in vitro.**

HUVECs were treated with 10 ng/mL rhVEGFA for 24 h, and untreated cells served as controls.
**(A-B)** Representative EdU proliferation assay images of HUVECs (A) and quantification (B).
**(C-D)** Representative tube formation images (C) and quantification of branch points (D).
**(E-F)** Representative wound healing migration images of HUVECs (E) and quantification (F).
**(G-H)** Representative transwell migration images of HUVECs (G) and quantification (H).

All data are presented as the mean ± SD from 6 independent experiments. **P < 0.01.


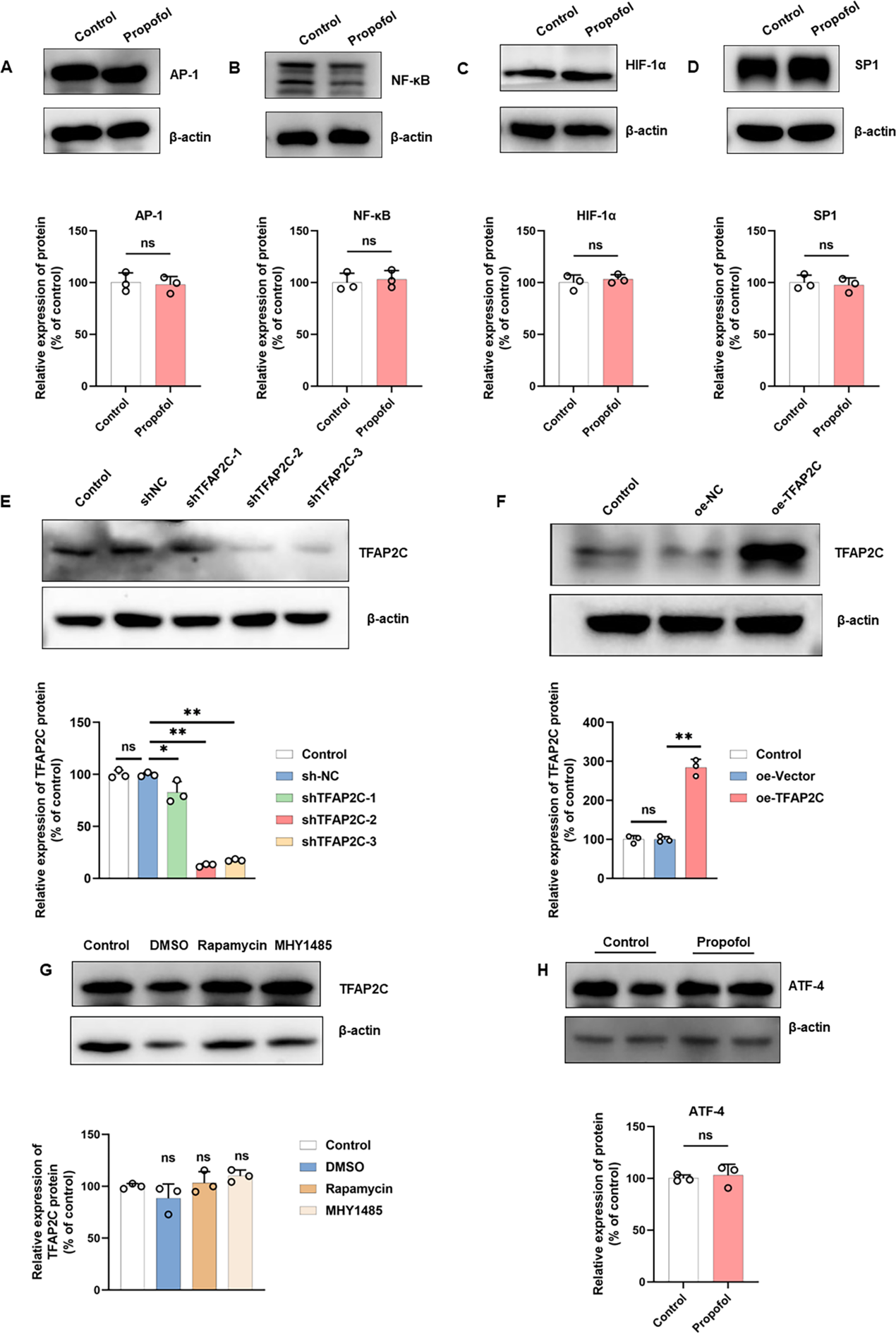


**Fig. S3 Protein expression levels of classical and potential transcriptional regulators of VEGFA.**

**(A)** Western blot analysis of AP-1 expression in HUVECs treated with 50 μM propofol.
**(B)** Western blot analysis of NF-κB expression in HUVECs treated with 50 μM propofol.
**(C)** Western blot analysis of HIF-1α expression in HUVECs treated with 50 μM propofol.
**(D)** Western blot analysis of SP1 expression in HUVECs treated with 50 μM propofol.

**(E)** Western blot analysis of TFAP2C expression in HUVECs transfected with NC (Control), empty vector (shNC), or TFAP2C knockdown (shTFAP2C).
**(F)** Western blot analysis of TFAP2C expression in HUVECs transfected with NC (Control), empty vector (oe-NC), or TFAP2C overexpression (oe-TFAP2C).
**(G)** Western blot analysis of TFAP2C expression in HUVECs treated with the mTOR inhibitor (50 nM Rapamycin) or activator (10 μM MHY1485).
**(H)** Western blot analysis of ATF-4 expression in HUVECs treated with 50 μM propofol.
All data are presented as the mean ± SD from 3 independent experiments. *P < 0.05, **P < 0.01.
