# Supplementary material for: Propofol attenuates angiogenesis by activating endoplasmic reticulum stress to suppress TFAP2C-driven VEGFA transcription
Source: Apoptosis. 2026 Jan 12;31(1):42. doi: 10.1007/s10495-025-02214-w (PMC12795951; doi:10.1007/s10495-025-02214-w)
Supplement: Supplementary file 2 — Supplementary Material 2 [file 10495_2025_2214_MOESM2_ESM.docx]

**Supplemental Table S1** Primers used for qRT- PCR.

| Genes | Forward | Reverse |
| --- | --- | --- |
| VEGFA | AGGGCAGAATCATCACGAAGT | AGGGTCTCGATTGGATGGCA |
| TFAP2C | TCAGTCCCTGGAAGATTGTCG | CCAGTAACGAGGCATTTAAGCA |
| GAPDH | GGGAGCCAAAAGGGTCATCA | TGATGGCATGGACTGTGGTC |

**Supplemental Table S2** The list of antibodies.

| Protein name | Application | Dilution | Company | Catalogue number |
| --- | --- | --- | --- | --- |
| VEGFA  VEGFR2  p-VEGFR2  TFAP2C  PERK  p-PERK  eIF2α  p-eIF2α  Puromycin  AP-1  NF-κB  HIF-1α  SP1  β-actin  VEGFA  p-VEGFR2  TFAP2C  Puromycin  VEGFA  CD31  TFAP2C | WB  WB  WB  WB  WB  WB  WB  WB  WB  WB  WB  WB  WB  WB  IF  IF  IF  IF  IHC  IHC  CHIP | 1/5000  1/1000  1/1000  1/1000  1/1000  1/1000  1/1000  1/1000  1/2000  1/1000  1/5000  1/2000  1/5000  1/5000  1/200  1/100  1/100  1/100  1/500  1/100  10 ug/ml lysate | ProteinTech  ProteinTech  CST  ProteinTech  Zenbio  Zenbio  Zenbio  Zenbio  Abclonal  ProteinTech  ProteinTech  ProteinTech  ProteinTech  ProteinTech  ProteinTech  CST  ProteinTech  ABclonal  ProteinTech  Abcam  ProteinTech | 19003-1-AP  26415-1-AP  2478T  14572-1-AP  R25331  340846  201137  R22946  A23031  10486-1-AP  80979-1-RR  20960-1-AP  21962-1-AP  27309-1-AP  19003-1-AP  2478T  14572-1-AP  A23031  66828-1-Ig  ab281583  14572-1-AP |

**Supplemental Table S3** Promoter sequence of VEGFA.

>5' Flanking sequence chromosome:GRCh38:6:43768184:43770183:1

TGCTCCTGGGGTGCTAGAGGCGCACAAGGAGGAAAGTTAGTGGCTTCCCTTCCATATCCCGTTCATCAGCCTAGAGCATGGAGCCCAGGTGAGGAGGCCTGCCTGGGAGGGGGCCCTGAGCCAGGAAATAAACATTTACTAACTGTACAAAGACCTTGTCCCTGCTGCTGGGGAGCCTGCCAAGTGGTGGAGACAGGACTAGTGCACGAATGATGGAAAGGGAGGGTTGGGGTGGGTGGGAGCCAGCCCTTTTCCTCATAAGGGCCTTAGGACACCATACCGATGGAACTGGGGGTACTGGGGAGGTAACCTAGCACCTCCACCAAACCACAGCAACATGTGCTGAGGATGGGGCTGACTAGGTAAGCTCCCTGGAGCGTTTTGGTTAAATTGAGGGAAATTGCTGCATTCCCATTCTCAGTCCATGCCTCCACAGAGGCTATGCCAGCTGTAGGCCAGACCCTGGCAAGATCTGGGTGGATAATCAGACTGACTGGTCCCACTCTTCCCACAGGCCTCAGAGCCCCAACTTTGTTCCCTGGGGCAGCCTGGAAATAGCCAGGTCAGAAACCAGCTAGGAATTTTTCCAAGCTGCTTCCTATATGCAAGAATGGGATGGGGCCTTTGGGAGCACTTAGGGAAGATGTGGAGAGTTGGAGGAAAAGGGGGCTTGGAGGTAAGGGAGGGGACTGGGGGAAGGATAGGGGAGAAGCTGTGAGCCTGGAGAAGTAGCCAAGGGATCCTGAGGGAATGGGGGAGCTGAGACGAAACCCCCATTTCTATTCAGAAGATGAGCTATGAGTCTGGGCTTGGGCTGATAGAAGCCTTGGCCCCTGGCCTGGTGGGAGCTCTGGGCAGCTGGCCTACAGACGTTCCTTAGTGCTGGCGGGTAGGTTTGAATCATCACGCAGGCCCTGGCCTCCACCCGCCCCCACCAGCCCCCTGGCCTCAGTTCCCTGGCAACATCTGGGGTTGGGGGGGCAGCAGGAACAAGGGCCTCTGTCTGCCCAGCTGCCTCCCCCTTTGGGTTTTGCCAGACTCCACAGTGCATACGTGGGCTCCAACAGGTCCTCTTCCCTCCCAGTCACTGACTAACCCCGGAACCACACAGCTTCCCGTTCTCAGCTCCACAAACTTGGTGCCAAATTCTTCTCCCCTGGGAAGCATCCCTGGACACTTCCCAAAGGACCCCAGTCACTCCAGCCTGTTGGCTGCCGCTCACTTTGATGTCTGCAGGCCAGATGAGGGCTCCAGATGGCACATTGTCAGAGGGACACACTGTGGCCCCTGTGCCCAGCCCTGGGCTCTCTGTACATGAAGCAACTCCAGTCCCAAATATGTAGCTGTTTGGGAGGTCAGAAATAGGGGGTCCAGGAGCAAACTCCCCCCACCCCCTTTCCAAAGCCCATTCCCTCTTTAGCCAGAGCCGGGGTGTGCAGACGGCAGTCACTAGGGGGCGCTCGGCCACCACAGGGAAGCTGGGTGAATGGAGCGAGCAGCGTCTTCGAGAGTGAGGACGTGTGTGTCTGTGTGGGTGAGTGAGTGTGTGCGTGTGGGGTTGAGGGCGTTGGAGCGGGGAGAAGGCCAGGGGTCACTCCAGGATTCCAATAGATCTGTGTGTCCCTCTCCCCACCCGTCCCTGTCCGGCTCTCCGCCTTCCCCTGCCCCCTTCAATATTCCTAGCAAAGAGGGAACGGCTCTCAGGCCCTGTCCGCACGTAACCTCACTTTCCTGCTCCCTCCTCGCCAATGCCCCGCGGGCGCGTGTCTCTGGACAGAGTTTCCGGGGGCGGATGGGTAATTTTCAGGCTGTGAACCTTGGTGGGGGTCGAGCTTCCCCTTCATTGCGGCGGGCTGCGGGCCAGGCTTCACTGAGCGTCCGCAGAGCCCGGGCCCGAGCCGCGTGTGGAAGGGCTGAGGCTCGCCTGTCCCCGCCCCCCGGGGCGGGCCGGGGGCGGGGTCCCGGCGGGGCGGAGCCATGCGCCCCCCCCTTTTTTTTTT
